# A “Good Life” for Dairy Cattle: Developing and Piloting a Framework for Assessing Positive Welfare Opportunities Based on Scientific Evidence and Farmer Expertise

**DOI:** 10.3390/ani12192540

**Published:** 2022-09-22

**Authors:** Jessica E. Stokes, Elizabeth Rowe, Siobhan Mullan, Joy C. Pritchard, Rachel Horler, Marie J. Haskell, Cathy M. Dwyer, David C. J. Main

**Affiliations:** 1 School of Agriculture, Food and Environment, Royal Agricultural University, Cirencester GL7 6JS, UK; 2 Farm Animal Welfare, School of Veterinary Sciences, University of Bristol, Bristol BS40 5DU, UK; 3 UCD Veterinary Sciences Centre, University College Dublin, Belfield, D04 V1W8 Dublin, Ireland; 4 Maundrils Farm, West Huntspill, Highbridge TA9 8QS, UK; 5 Animal Behaviour & Welfare, Animal & Veterinary Sciences, Scottish Rural University College, Edinburgh EH25 9RG, UK

**Keywords:** dairy cattle, animal welfare, positive welfare, quality of life, animal welfare assessment, animal welfare policy, farmer wellbeing

## Abstract

**Simple Summary:**

There is increasing appetite to understand how we can provide quality of life to farm animals. A framework to evaluate positive welfare opportunities for dairy cattle was developed using a participatory approach where farmer’s recommendations were integrated into a scientific framework and piloted on farm by vets. When provided with the opportunity to collaborate, farmers and scientists broadly agree on what constitutes “a good life” for dairy cattle and worked together to develop an assessment framework. Farmers did not agree equally on the value of each positive welfare opportunity. However, farmers supported positive welfare assessment as a means of recognition and reward for higher animal welfare, within existing farm assurance schemes, and to justify national and global marketing claims of higher animal welfare.

**Abstract:**

On-farm welfare assessment tends to focus on minimising negative welfare, but providing positive welfare is important in order to give animals a good life. This study developed a positive welfare framework for dairy cows based on the existing scientific literature which has focused on developing positive welfare indicators, and trialled a participatory approach with farmers; refining the framework based on their recommendations, followed by a vet pilot phase on farm. The results revealed that farmers and scientists agree on what constitutes “a good life” for dairy cattle. Farmers value positive welfare because they value their cows’ quality of life, and want to be proud of their work, improve their own wellbeing as well as receive business benefits. For each good life resource, the proportion of farmers going above and beyond legislation ranged from 27 to 84%. Furthermore, barriers to achieving positive welfare opportunities, including monetary and time costs, were not apparently insurmountable if implementation costs were remunerated (by the government). However, the intrinsic value in providing such opportunities also incentivises farmers. Overall, most farmers appeared to support positive welfare assessment, with the largest proportion (50%) supporting its use within existing farm assurance schemes, or to justify national and global marketing claims. Collaborating with farmers to co-create policy is crucial to showcase and quantify the UK’s high welfare standards, and to maximise engagement, relevance and uptake of animal welfare policy, to ensure continuous improvement and leadership in the quality of lives for farm animals.

## 1. Introduction

Society values farm animal quality of life: consumer awareness, willingness to pay and demand for higher welfare products is increasing globally [1,2]. Many consumers want to buy products from animals that have had positive welfare experiences [3] and farmers [4], certifiers and suppliers [5] want to demonstrate they can provide these products. The UK government has instructed DEFRA to explore the development of public good payments for farmers achieving higher welfare post Brexit [6].

Despite a movement in the last two decades within the animal welfare science community to advance the investigation of positive welfare [7] and a decade since the aspiration of providing farm animals with a ‘good life’ (where positive experiences outweigh negative ones) was proposed [8], the measurement of positive welfare systematically on-farm in the UK has not been adopted [9]. A framework for recognising and championing existing positive welfare opportunities on farm, as well as a mechanism for rewarding practices that promote positive welfare, could be a novel approach to animal welfare policy [10].

Dairy farmers are a primary stakeholder in their cow’s wellbeing, and in recent years a few farmers have taken ownership over growing societal concerns in animal welfare, taking the lead to provide innovative solutions to the industry’s ethical dilemmas over separating cows and calves and an increasing move towards zero-grazing [4,11,12]. However, there is no standardised means of recognising these and other farmers who are providing positive welfare opportunities, as stewards and leaders in farm animal welfare. Furthermore, there is relatively little known about the wider farming community’s attitudes and perspectives of positive welfare [13,14].

Several approaches for recognising positive welfare have been proposed by the scientific community; for example: grading resources which provide opportunities for positive welfare [9,15,16,17]; measuring pleasurable behaviours directly such as play [18,19,20,21,22] and observing body language and indicators of emotion [23,24,25,26]. Although animal-based measures of positive welfare—those that specify an animal’s state [27]—provide a direct assessment of positive welfare, they are yet to be well validated and standardised, whereas resource-based measures are more practical and considered easier for farmers to accept and use [7].

A quality of life framework based on resource provision was proposed by the Farm Animal Welfare Council [8], which suggested four opportunities that characterise a ‘good life’ for farmed animals. These are the opportunities for comfort, pleasure, interest and confidence [8]. Edgar et al. [16] added a fifth opportunity for a ‘healthy life’, in order to achieve a balance between animals being healthy and having the resources they ‘want’ (are highly motivated to obtain): two factors underpinning good welfare [28]. This research team developed a ‘good life’ framework for laying hens based on resources needed to provide hens with these five opportunities, according to scientific literature and expert knowledge. Resources were ranked to create three levels of increasing positive welfare (‘Welfare +’, ‘Welfare ++’, ‘Welfare +++’) [16].

Using the work described above as a template, a positive framework for dairy cattle was drafted based on a review of the existing literature to identify what resources dairy cows’ value based on the good life concepts of comfort, pleasure, interest, confidence and a healthy life (see Table S1). The framework was designed to quantify increasing positive welfare opportunities in terms of three tiers: Welfare +, Welfare ++, and Welfare +++, above and beyond legislation and welfare codes in the UK.

The research team wished to build on this draft framework by collaborating with farmers to further develop the resource tiers based on farmer knowledge and experience. Working with farmers is essential to deliver relevant and palatable research and policy outcomes that will directly affect end users [29]. Integrating farmers in academic research not only utilises their expertise, but aims to create buy-in for the end result of the research, and provide an understanding of the potential barriers to, and drivers for, the successful uptake of research outcomes. It also gives farmers an opportunity to showcase best practice and be in the driving seat of research and innovation. Therefore, use of a facilitation process in farmer focus groups was hypothesized to engage farmers with the concept of the research and embed their ideas and practices within the positive welfare framework, which would be taken forward to trial on a representative sample of UK dairy farms.

The aim of this study was five-fold: (1) to develop a framework for providing positive welfare opportunities for dairy cows, basing resource provisions on a review of the scientific literature; (2) to trial a novel participatory approach to consulting farmers on the positive welfare framework; (3) to refine the framework based on farmers recommendations; (4) to investigate farmers attitudes towards positive welfare and use of the framework; and (5) to pilot the positive welfare framework as an on-farm assessment tool, and seek farmer feedback on its value and potential uses, as well as any barriers to and potential incentives for farmers providing positive welfare opportunities for dairy cows.

## 2. Materials and Methods

This paper presents the development of a positive welfare framework for dairy cattle (Table S1) using the policy develop process and outcomes represented in four steps: literature review, farmer consultation development; farmer consultation; and engagement with veterinary practitioners to pilot positive welfare framework (see Table S2). As there is a growing policy interest in agricultural research and innovation generated using a multi-actor co-design approach including key stakeholders (research scientists, farmers and veterinarians) that fosters a high level of farmer engagement from the conceptualization phase [29], a participatory policy development process was adapted from a previous project [29] and applied during the current study. The full details of the policy development process are found in Table S2.

### 2.1. Literature Review

At the beginning of this study, a literature review was carried out to develop the evidence-base and good life resources for each opportunity proposed in the framework. The framework consists of 14 resource needs for dairy cattle (for example comfort by choice of physical environment) categorized under five good life opportunities [8] of comfort, pleasure, confidence, interest and healthy life (see Table S1). For each resource need, a scale of increasing welfare opportunity (above and beyond law and codes of practice) was developed and described as Welfare +, Welfare ++ and Welfare +++. The framework was developed to assign a welfare category (Welfare +, Welfare ++ and Welfare +++) for each resource need based on an inspection of physical resources, the on-farm environment and proactive management activities (see Table S1). In addition, as part of the literature review each potential resource need to achieve each good life opportunity of comfort, pleasure, interest, confidence and a healthy life was evaluated with regard to its validity and reliability in increasing cow welfare, and the feasibility of providing the necessary resources required to fulfill each opportunity was assessed (see Table S1).

### 2.2. Developing a Collaborative Participatory Approach

A team workshop including all research institutes collaborating on this project (Royal Agricultural University, University of Bristol and Scotland’s Rural College) was held in May 2016 to adapt and apply an existing participatory approach for the purpose of consulting with dairy farmers on the positive welfare framework [29]. The authors set the intention to use a series of in-depth focus group meetings with two core groups in the surrounding dairy producing areas of each research institute (South West and South East of England, and Scotland). The size of the focus group (2–10 farmers) was agreed to facilitate a variety of views emerging from the group while ensuring discussion and group exercises were feasible, and each farmer had an opportunity to contribute fully. Questions and exercises to capture the views and practices of farmers and facilitate discussion were drafted by the team, and finalised by the first author who was responsible for facilitating the series of focus group meetings. The policy process steps to develop a positive welfare framework in practice are outlined in Table S2.

### 2.3. Recruitment of Dairy Farmers

The main focus group of dairy farmers was recruited in September 2016 via the leading industry consultant delivering discussion group meetings throughout the South West and East of England. An email was sent out to existing meeting members outlining the aims of the project and requesting participation. Eight farmers volunteered to participate: four women and four men managing one small rare breed herd, one free range dairy (guaranteed 180 days access to pasture), two organic (on average 215 days at pasture), two traditional systems (access to pasture during the summer grazing season and housed during the winter), two restrictive grazing systems (access to pasture 2–4 h a day from spring through to late autumn) and one continuously housed (no access to pasture) herd across Somerset and Gloucestershire.

### 2.4. Dairy Farmer Focus Group

The main focus group participated in three in-depth meetings (2–3 h) between November 2016 and February 2017, to develop the framework. Participants were guaranteed anonymity and agreed to be audio recorded (using a Dictaphone) at each meeting. A further two dairy farmers in the Scottish Borders were consulted in September 2017 during one meeting where the recommendations of the main focus group were shared and they were invited to provide additional input.

#### 2.4.1. Meeting One—Positive Welfare Definitions, Values and Motivations

Following a general introduction, farmers took part in two exercises in turn, submitting written ideas in relation to the following questions for subsequent discussion, consolidation and write up by the facilitator (see Table S1):

- What is the value of positive welfare? What are the benefits of a good life for your dairy cows?
- How do you define positive welfare? What is a good life for your cows?

#### 2.4.2. Meetings Two and Three—Developing the Positive Welfare Framework

Between meetings one and two, the facilitator collated the farmers’ ideas alongside the previously drafted framework (see Table S1). At meetings two and three, the group’s ideas, practices and aspirations for positive welfare assessment gathered during meeting one were presented back to the group alongside the relevant opportunity in the framework. The facilitator then led a discussion to hone down and embed the farmer’s ideas one by one, into the relevant opportunity, until the content and levels of criteria was broadly agreed by the group. This involved an iterative discussion around the value of each opportunity as well as the perceived practicality, acceptability, uptake by other dairy farmers, and the costs and benefits of opportunities which opposed existing conventional practices. Where the group deemed it necessary, the criteria for achieving an entry level (Welfare +) was adapted to make it as accessible as possible, while ensuring there was a distinct step up from the existing baseline legislation and welfare codes. To this end, in a few cases, the group recommended making the entry level harder than originally stated in the preliminarily scientific draft. After agreeing the content for each positive welfare opportunity, the facilitator finally asked the group to discuss the following question:

- What would incentivise you, other farmers and the sector to deliver positive welfare?

#### 2.4.3. Meeting Four with Scottish Dairy Farmers

A 4th meeting was arranged by Scotland’s Rural College (SRUC) with Scottish dairy farmers to present and discuss the input gained from the focus group. The framework was sent to the participants in advance. The meeting was facilitated by members of the research team (JS + MH) and was recorded and transcribed verbatim. Any amends and ideas in terms of value and use of the framework from this group were then integrated into the working draft and results.

### 2.5. On-Farm Pilots

XLVets (https://www.xlvets.co.uk/, accessed on 10 September 2022), a community of independent veterinary practices, partnered with the research team to carry out this final stage of the project. All participating veterinary practices contributed their time free of charge due to the practice valuing engagement in, and advancement of, on farm welfare standards. A member of the research team (DM) conducted a training session with participating vets on the Royal Agricultural University’s (RAU) farm, to demonstrate how to assess dairy farms against the positive welfare framework. Veterinary practices nominated dairy farmer clients as participants for the research, and were sent participant information sheets explaining the research by the vet collecting the on farm data. Farmers who volunteered to participate in the study signed a consent form, and were informed that they could withdraw from the research at any time. The study methodology was reviewed and approved by the RAU Ethics Research Committee (Approval number 2019.0004).

Thirty-four farmers were recruited to the study. Farms were visited by a vet from a participating veterinary practice between March and August 2019. Each visit lasted approximately 1 h. Half of this time was allocated for the farm assessment conducted by the vet using the positive welfare framework. This entailed the vet using the framework to record the presence or absence of each resource requirement for each welfare level (welfare +, welfare ++, welfare +++) for each positive welfare opportunity. The other half of the visit was allocated for a farmer interview, conducted by the vet using a questionnaire that asked about the farmer’s views on the framework; these were:

- Which good life opportunity(s) or resource(s) they valued, and those they did not consider valuable;
- How the framework should/could be used.

The following questions were asked pertaining to four of the good life opportunities in the framework: comfort by physical environment, interest by pasture choices, pleasure by play and positive social interactions, and pleasure by maintenance of the cow-calf bond. These four opportunities were chosen because during the initial consultation with farmers through focus groups, these opportunities were either the most valued (comfort by physical environment and pleasure by play and positive social interactions) or the cause of most debate due to the differentiation they posed between dairy systems (interest by pasture choices and pleasure by maintenance of the cow-calf bond). Questions asked about these four opportunities were:

- If these opportunities for positive welfare were not being achieved on their farm, the reasons why;
- The estimated monetary cost of achieving these opportunities for positive welfare on their farm;
- Minimum annual government payments they would accept to implement changes required to achieve these opportunities for positive welfare on their farm;
- How likely they would be to provide these opportunities for positive welfare if given government funding to cover the full costs of implementing changes required.

The following descriptive data about the farms were also collected via the questionnaire:

- Farm location;
- Herd size;
- Calving interval;
- Milk sold;
- Days grazed;
- System type (conventional, organic, pasture fed);
- Farm assurance status.

The questionnaire was a mixture of open questions, multiple choice questions and rating scales. A copy of the questionnaire can be obtained from the corresponding author.

### 2.6. Data Analysis

The audio recordings from the farmer focus groups were transcribed verbatim. The transcripts and written exercises were analysed using NVivo 11 to draw out themes associated with the farmer’s values, definitions and practices of positive welfare. The focus group participants’ motivations to take part are summarised using quotes, and their value of positive welfare are consolidated using a word cloud created via NVivo 11. Changes to the content or levels of criteria for each positive welfare opportunity are summarised, in order to demonstrate the outcomes of the consultation process (Table S1). Descriptive data and qualitative responses to the questionnaire are given for the on-farm piloting of the framework by XLVets. For each positive welfare opportunity, the percentage of farmers achieving at least one resource requirement was calculated for each welfare level (welfare +, welfare ++, welfare +++), expressed as a percentage of the maximum possible achievement of meeting all resource requirements for all welfare levels in that positive welfare opportunity. This equation is given below:

% farmers achieving at least one resource requirement for the given welfare level in the given positive welfare opportunity = Sum of farmers achieving any resource requirement in the given welfare level/(total number of farmers x total number of resource requirements in the given positive welfare opportunity).

## 3. Results

### 3.1. Thematic Analysis of Focus Groups

The major themes which emerged from the focus groups are presented. Dairy farmer’s motivations to engage in developing positive welfare policy, as well as their values of positive dairy welfare is summarised and supported using indicative quotes. Dairy farmer’s definitions and practices were integrated into the positive welfare framework (see Table S1). Finally, themes on how to incentivise other farmers to engage in the positive welfare framework is presented and consolidated using indicative quotes.

### 3.2. Motivation to Engage in Positive Welfare Policy Development

All dairy farmers reported three main drivers for taking part in the focus groups:

Their attitude towards providing positive welfare for their livestock:

“It’s mainly my husband and me who does all the work and we have always been interested in positive welfare and taking that extra time and detail with our cows”.

To have a say in the future:

“I feel you can’t complain about standards being imposed if you don’t take the chance to have an input”.

A desire to fulfil public perception:

“We milk 200 cross breeds and we’ve also joined Neil Derwent’s free-range dairying brand. I think the public perception is that cows do graze and they’d be surprised to hear that some cows don’t graze. I’m interested in that”.

One further motivation was highlighted by a member of the focus group:

“I have a particular responsibility within our supply chain for managing and improving animal health and welfare”.

### 3.3. Farmers Values of Positive Dairy Cattle Welfare

**Figure 1:**
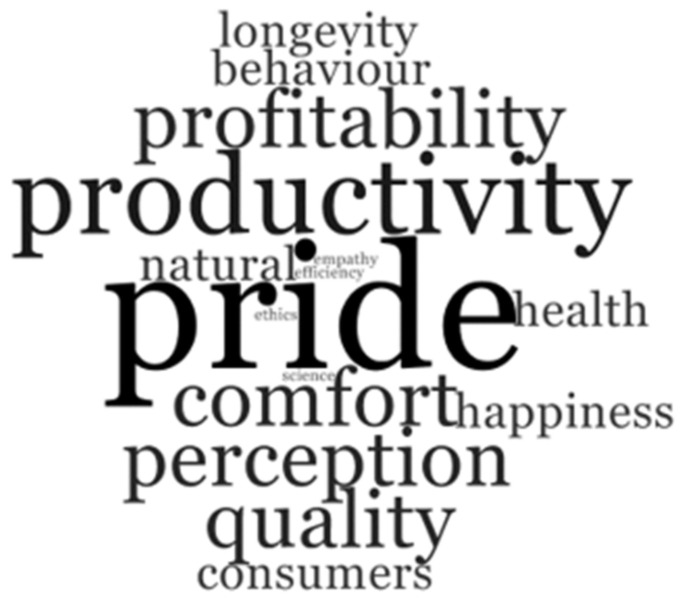
Word cloud representing farmer focus group values of positive welfare.

Understanding farmers’ values and farmers taking ownership over policy development is pertinent if that policy is going to reflect what is happening on the ground and is to be adopted more widely. Values reflect what people think is important to them and are a rationale for why actions are taken. The values of positive welfare reported by the dairy farmers in focus group meeting one is illustrated in Figure 1 as a visual representation of the number of times they were articulated by farmers. The bigger the word, the more times it was voiced by farmers in the group. These values are expanded upon below.

#### 3.3.1. Value One: Farmer Pride and Wellbeing

One of the most expressly reported values and motivation for delivering positive animal welfare was the farmers’ pride and wellbeing. The main drivers that were reported to be behind this value were empathy, their sense of responsibility to rear happy, healthy animals which have more positive than negative welfare experiences, and the feeling of wanting to protect their cows. Farmers valued animals being in a positive state of welfare because it is inextricably linked to their own wellbeing:

“I was so upset about those cows because someone had upset them. Do you know it affected me for the rest of the day? There is something about being protective of your cows. That relationship between you and your cows, that when they have a negative experience, you have a negative experience”.

“I think the cows know. I think they sense a lot of what we feel. When they are stressed you are stressed. And I think we can stress them by being stressed ourselves”.

Related to the farmer’s wellbeing, their farm staff morale and job satisfaction was deemed an important driver for providing positive welfare:

“Staff morale is key. Staff don’t like it if you have a non-content cow, and if something happens to a cow it really knocks them. It’s just easier and more satisfying if it never happens in the first place. Happy and healthy animals are easier to manage”.

#### 3.3.2. Value Two: Quality of Life for Dairy Cattle

All dairy farmers during the focus group meetings reported and agreed on the importance of positive welfare for the cow’s own quality of life. The main reported drivers were wanting happy cows, the perception that happier cows live a longer life; that it is inherently good for a cow to be able to express her natural behaviour, and that valuing and delivering positive welfare assures that cattle are comfortable. In one farmer’s words summarising the group’s ideas:

“You value positive welfare for the cows themselves very highly, in terms of health, happiness, comfort and behaviour but also the fact that happy cows live longer. It’s good for cows’ quality of life”.

#### 3.3.3. Value Three: Health and Productivity of Dairy Cattle

All dairy farmers in the focus group reported valuing positive welfare for the cow’s own health and productivity (see Figure 1). The main drivers behind this were: less illness in contented cows, improved quality and quantity of milk, better immunity, improved productivity and improved overall performance. Two farmers summed this up in their own words:

“Positive welfare means better immunity and less illness in contented cows. Positive welfare means better quality and quantity of milk”.

“As much as we appear to love our cows we are all business people. We are milking cows to earn a living and if the cows aren’t healthy they are not productive, and we don’t earn a living, so we won’t be doing it for very long”.

#### 3.3.4. Value Four: Consumer Perception and Market Premium

Finally, the focus group suggested that positive welfare was highly valuable as a marketing and communication tool to improve consumer perception, demand and return for higher welfare products. For example:

“You’ve got basic productivity and then you’ve got the value of that product and what the consumer will pay for it and positive welfare feeds into that”.

“Positive welfare is really important for customers, consumers and the public perception of dairying”.

“I think the other thing we haven’t put in there is costs, because you know negative welfare experiences cost more. There may also be a cost implication for positive welfare. There is a positive cost where you might get more for your milk”.

### 3.4. Defining Positive Welfare

All suggestions given by the farmer focus group on how to define positive welfare under each of the positive welfare opportunities are given in Table S1. In summary, when farmers were asked how they define a good life for cows, they came up with resources and opportunities that all related to the same opportunities that were highlighted by reviewing the scientific literature, with two exceptions as follows. Firstly, farmers did not include keeping dairy cows and calves together in their unprompted suggestions for defining positive dairy cattle welfare. Secondly, farmers suggested the additional opportunity of providing cows with comfort by the opportunity for milking choices, which had not been included following the literature review.

### 3.5. Collaborative Development of the Positive Welfare Framework

During meeting 2 and 3, the focus group reviewed the previously drafted positive welfare framework (Table S1) and proposed amendments that are described in detail for each opportunity in the supplementary table (Table S1). There were also several generic changes proposed to make the framework more ‘user-friendly’ by streamlining and simplifying the original draft based on the feedback from the group. Therefore, the wording for law and code were removed, along with the scientific references.

### 3.6. Incentivising Engagement in Positive Dairy Welfare

The farmer focus groups suggested that incentivising engagement of other farmers with the positive welfare framework would depend on costing out the benefits of delivering each of the positive welfare opportunities and communicating this to farmers and policy makers. This would add value to the farmers, and provide evidence to policy makers of the value of paying farmers to employ the more expensive opportunities as public goods:

“Costing it out financially is the way we need to go—costing out the cost benefits of animal welfare. It’s really policy makers you have to convince with this because they are the ones to decide how the taxes are used”.

A consultation with consumers in the market place to establish which positive welfare opportunities were valued by society was also recommended:

“The positive publicity of welfare and public perception is key. We need to consult the public on what matters to them”.

This could feed into a market or government incentive scheme which could support farmers to transition towards the most highly valued opportunities that require substantial investment and/or a substantial change in mind-set.

The main incentives highlighted by farmers were to stay ahead of the game with regards to animal welfare and use an evidence-base like this framework in order to make valid animal welfare claims to their customers.

“Evidence base is absolutely. It is fundamental. Just to defend yourself in the future if you are going to make claims”.

### 3.7. On-Farm Piloting of the Positive Welfare Framework

#### 3.7.1. Farm Descriptive Data

For the piloting of the positive welfare framework by XLVets, there were missing descriptive data for five of the 34 farms (15%) in the study. Descriptive figures are expressed as percentages of the 29 farms for which data were available.

Most farms (n = 20, 69%) were in the South West of England: five in Somerset, four in Devon, four in Dorset, four in Gloucestershire and three in Wiltshire. Outside the South West, four farms (14%) were in Derbyshire, two (7%) in Oxfordshire, two in Worcestershire, and one (3%) in Nottinghamshire. The majority of farms (n = 23, 79%) were conventional, four (14%) were organic, and four described themselves as Pasture-Fed (grazing-based systems where the majority, but not necessarily the entirety, of feed is grass; of these four pasture-fed farms, two also classed themselves as conventional). All 29 farms were Red Tractor certified; of these, three (10%) were also assured by Soil Association, two (7%) by Arlagården, two by Tesco, one (3%) by Organic Farmers and Growers, one by RSPCA Assured, and one by Sainsbury’s. No farms were certified by Pasture for Life, which requires 100% of the cows’ diet to be grass/forage for Pasture-Fed systems.

Herd size ranged from 80 to 2100 cows (median = 200), although the largest figure was an outlier; if excluded the range was 80–390 cows (median = 200). Milk sold per cow per year ranged from 4300–12,000 L (median = 9000 L); one farmer gave this figure as a range, the median of which was taken as the data point. Number of days grazed ranged from 0–365 days (median = 180 days; where a range was reported, the median was taken as the data point, and where a minimum number of days grazed was reported, this minimum figure was used). Six farms were zero-grazing. Two farms grazed low yielding cows (including those in mid to late lactation, confirmed pregnant, or with high body condition score) between 180–200 days, but high yielding cows (including those in early lactation) for 0 days. Four farms (12%) carried out spring calving, 13 farms (38%) block calving, and 16 (47%) calved all year round.

#### 3.7.2. Farm Assessment of Positive Welfare Opportunities

There were missing farm assessments for five of the 34 farmers in the study, leaving a total of 29 farms for this section of the analysis.

Across 406 (29 × 14) combinations of farms and positive welfare opportunities, 34% of farms achieved Welfare +, 22% of farms achieved Welfare ++, and 4% of farms achieved Welfare +++. These data are shown in Table 1.

#### 3.7.3. Perception of Positive Welfare Opportunities

There were missing interview responses for seven of the 34 farmers in the study, leaving a total of 27 farms for this section of the analysis. Table 2 displays the positive welfare opportunities or resources most valued by farmers for which there are data for this part of the interview (n = 26); Table 3 displays those which farmers (n = 22) reported not to value (farmers could give more than one answer so percentages do not sum to 100).

#### 3.7.4. Reasons for Not Achieving Positive Welfare Opportunities

Table 4 illustrates reasons given by farmers as to why they were not (if they were not) achieving each welfare level for the four positive welfare opportunities in the questionnaire (comfort by physical environment, interest by pasture choices, pleasure by play and positive social interactions, and pleasure by maintenance of the cow-calf bond). Numbers of farmer responses are given as denominators in the table, as data were not captured for all 27 farmers. Table 5 displays reasons given by farmers other than those offered by multiple choice in the questionnaire.

#### 3.7.5. Likelihood of Achieving Positive Welfare Opportunity with Government Funding

Table 6 shows the likelihood of farmers making the changes required to meet each welfare level of each of the four good life opportunities if they were fully compensated for the costs required to make these changes. Not all farmers gave answers to this section of the questionnaire; number of responses are given as denominators in the table.

#### 3.7.6. Framework Use

There were missing data for the question on how farmers would like to see the framework used for one farm. Of the 26 dairy farmers for which data are available, 32% recommended the framework to justify national and global marketing claims of UK higher animal welfare; 32% supported its use within existing farm assurance schemes; 22% saw its use as part of a grants scheme for capital expenditure or training associated with enhanced animal welfare, and 14% of farmers recommended its use within a government funded animal welfare stewardship scheme.

During the on-farm pilot, one farmer suggested that including the positive welfare framework in farm assurance schemes was an opportunity to inform customers of good welfare provision, whilst another arguing against its inclusion believed that it might be difficult to remove auditors’ emotions from the process, as the assessment currently appeared too subjective.

Reasons for support of using the framework in a government-funded stewardship scheme included ensuring that all farmers achieved a minimum level of positive welfare for their animals whilst enabling payment for farmers that go beyond this; in addition, one farmer believed that such a scheme could enable grants for buildings and infrastructure required to increase positive welfare opportunities. Another supported this suggestion by arguing that providing resources for positive welfare would affect profitability, therefore such provisions would need to have monetary value through payments from a government scheme. However, two farmers reported that they were wary of government sanctions and involvement.

One farmer argued against mandatory introduction of the framework in any form because this would deny farmers the opportunity to develop their own markets for positive welfare products. Conversely, one farmer who was against all suggested uses of the framework argued that before any more standards are introduced, their benefits to farmers including contribution towards profitability need to be demonstrated; this farmer did not feel there was a value to the assurance scheme she/he was already audited to. A further concern about the use of the framework voiced by one farmer was that it would be very difficult to ‘police’ positive welfare provisions across a whole year.

## 4. Discussion

### 4.1. Fostering Farmer Engagement

The primary purpose of this study was to trial a novel participatory approach to developing positive welfare policy in collaboration with farmers. All farmers recruited actively engaged in the process from start to finish and dedicated up to a day of their time to do so free of charge. At the end of the three initial meetings, a framework was agreed for an on-farm trial and the farmers involved were willing to commit more time by hosting meetings to carry out the trialing process. We suggest this level of engagement and outcome provides support for developing a co-design approach to animal welfare policy development going forward.

With regard to positive welfare, this study provides evidence that these farmers place value here and are motivated to deliver this, because they recognise having healthy and happy cows is intrinsically valuable and motivating in itself, enabling pride, improving their own wellbeing, as well as receiving business benefits through improved productivity, and being able to market positive welfare to consumers.

Communicating pride is crucial at a time when the dairy industry is coming under increasing scrutiny for welfare-negative practices that are perceived to be out of step with changing societal expectations. Public awareness and demand for higher dairy cattle welfare products is increasing [1], and although consumers are willing to pay for increasingly higher welfare, they cannot satisfy their preferences for these products currently because of a lack of information [30]. A standardised and evidence-based means of assessing and communicating the positive welfare opportunities farmers are delivering for dairy cattle (and other farm species) is greatly needed [14]. This framework proposes an evidence-based mechanism, described by farmers as “fundamental”—to defend themselves in claims they make, and any made against them.

### 4.2. Evidence of Existing Positive Welfare

During the on-farm piloting phase conducted by their vets, it was shown that 34% of farms achieved a Welfare +, 22% of farms achieved a Welfare ++, and 4% of farms achieved a Welfare +++. This suggests that a considerable proportion of farmers in this study may be going above and beyond legislation and codes of recommendations for dairy cattle welfare. This is an initially promising result, in view of the applicability of such a framework as a national benchmark for higher animal welfare. Far fewer farmers provided any resources required to achieve the highest level of welfare (‘welfare +++’). Together these results indicate that this framework is attainable at the lower end and aspirational at the upper end, providing scope for a continuous improvement mechanism for positive welfare opportunities within the UK dairy herd, either for example within existing farm assurance schemes, and as part of a government led grants and payments by results scheme for animal welfare enhancements that are valued by the public and not delivered sufficiently by the market [31]. However, further research and data collection is needed at a national level to demonstrate to what extent this is reflective of the UK dairy herd as a whole. In addition, further work is needed to train assessors and standardise the assessment protocol and enable valid comparisons to be made.

### 4.3. Positive Welfare Value to Farmers

The positive welfare opportunity valued by the highest proportion of farmers during the on-farm piloting phase was ‘comfort’ (46%), with an additional two (8%) specifying physical comfort and a further two specifying thermal comfort. This was followed by ‘healthy life’ (42%), and then interest by pasture choices (31%). However, over a quarter of the farmers (27%) said that they valued all of the good life opportunities. Overall, this indicates a relatively positive attitude to the concept amongst farmers who were part of the wider pilot phase, especially with respect to comfort, health, and pasture access. Significantly, these results mirror findings from a recent survey which reported that the top three welfare attributes that concerned 2,054 UK dairy consumers were: access to grazing, health and welfare, and cow comfort [32].

### 4.4. Government Incentives for System Change

One of the most valuable opportunities by farmers is not attainable without major infrastructure changes. In the UK, the majority of cows are kept in cubicles, and above level ‘welfare +’ for comfort by physical environment specifies straw yards. Farmers reported favouring cubicles compared to straw yards in reducing the risk of mastitis, presumably through improved udder hygiene. There is scientific evidence to support this: a review revealed that the use of cubicles was associated with lower somatic cell counts in dairy cows [33]. However, the husbandry practices with the most consistent associations with mastitis were related to milking procedures, rather than housing or bedding [33]. Nonetheless, cow hygiene in loose housing remains a legitimate concern, as labour and bedding costs required to maintain cleanliness may be increased in this system. However, these barriers are not apparently insurmountable, as farmers reported they would be ‘very likely’ to achieve the opportunity for comfort if remunerated by the government for the cost of implementing straw yard systems (see Table 6). This demonstrates a willingness by these farmers to change major infrastructure where government and society perceive this as a public good.

Not all positive welfare opportunities were equally valued by farmers, both within the focus groups and the on-farm pilot. For example, during the development phase, farmers in the focus group agreed with the value and inclusion of the aspirational positive welfare opportunity for pleasure, by maintaining the cow-calf bond. However, when it came to on farm piloting, some of these farmers questioned the value and merit of including an opportunity which goes above and beyond the current management strategies of conventional dairy farms in the UK. Over half the farmers during the on-farm pilot phase (for which data were available) reported not to value this opportunity. This divergence between the focus group and pilot farmers may be explained by different attitudes and perceptions, as well as levels of engagement and therefore ownership in the development process. For example, the focus group volunteered to take part, and despite not practicing it themselves, felt that farmers who were achieving this opportunity should be recognised and rewarded due to both the significant benefit to dairy cow and calf welfare, and value to consumers and the public. Furthermore, the focus group had the opportunity to discuss the frameworks philosophy over a series of meetings: “something for everyone, not everything for everyone”. This can be seen to further highlight the importance of engaging all stakeholders at the infancy of policy development, in order to increase their ownership and uptake of the process. In contrast, after being scored on the framework by their vet, farmers during the pilot phase had 30 min to be introduced to the concept of positive welfare. In addition, it may not have been made clear that this framework is being proposed as a voluntary rather than a sanctioning mechanism. Nonetheless, these farmers reported concern that keeping calves with their dams will reduce their welfare by increasing the risk of injury and disease, and increasing distress at eventual separation. Farmers perceived that distress to both calf and dam is reduced by immediate removal, for which there is scientific support. A recent review comparing separation at 6–24 h of age with separation at 4–14 days of age concluded that almost immediate separation (within the first day of life) is less distressing to both dam and calf than separating after the first few days to two of weeks of life [34]. However, a study comparing separation at 25 days with 45 days of age [34] found increased indicators of distress in both cows and calves following earlier separation. Several studies have shown that total suckling duration per day decreases with age [35] as calves become less nutritionally dependent on the dam. In addition, behavioural observations demonstrated greater social independence at the older age, suggesting the earliest age to begin separation to avoid acute distress is 6–7 weeks [36].

The perception of farmers during the on farm pilot phase that near-immediate separation of calves from dams reduces the risk of injury and disease for both calf and cow is not supported by scientific evidence: “the scientific peer-reviewed literature on cow and calf health provides no consistent evidence in support of early separation” and therefore “does not support a recommendation of early dairy cow-calf separation on the basis of calf or cow health” [37]. The authors found that studies on calf immunity, mortality, scours and pneumonia did not find that early separation confers health or survival benefits, or controls Johne’s disease, and that suckling is protective against mastitis, indicating a health benefit to keeping calves with dams.

Furthermore, prolonged cow–calf contact beyond the first day of life benefited calf welfare in the long term [34]. As part of this systematic review [34], over 80% of relevant research papers report beneficial effects of extended cow–calf contact on social behaviour, such as increased social interaction, and 75% report reduced abnormal oral behaviours in calves, both during and after the suckling period [34]. In addition, 71% of papers report reduced stress and/or fear responses in calves experiencing prolonged contact with the dam. Thus, there are multiple positive welfare benefits to keeping cows and calves together (for at least 6 weeks to avoid acute distress), compared to removing calves within the first day.

In light of this review of the evidence and the farmer feedback, the requirements for ‘welfare +’ in the opportunity for pleasure by maintenance of the cow-calf bond were changed to separation within the first 24 h of birth, whilst emphasising the need for calves to be kept in stable groups. The requirement for keeping dams and calves together for two weeks in level ‘welfare ++’ (added following the focus group consultation) was changed to 6 weeks to reflect the evidence base. This illustrates the iterative process of the development of a positive welfare framework, through collaboration between stakeholders, as well as incorporating evidence from new studies and literature reviews as they become available.

For level ‘welfare +++’, which prescribes keeping the calf with its dam until natural weaning, cost and time became the overriding hinderance. Farmers who were part of the on-farm pilot did see the benefit of keeping calves with mothers until naturally weaned, but stated this is completely impractical and uneconomic within the current conventional system. Dairy farmers have come up against most criticism by animal welfare advocates for separating calves from cows, and 15% of UK consumers reported wanting calves to stay with cows for longer in a recent survey [32]. Consumer confidence in dairy has been found to decline having visited a University farm mostly because of cow-calf separation [38]. Furthermore, a study in Germany identified that a 1/3rd of consumers following a vegan diet may be open to forms of animal agriculture guaranteeing animal welfare standards going beyond current practices [39].

### 4.5. Agreement between Scientists and Farmers

It is encouraging that only one farmer reported that opportunities for positive welfare was a new idea to them. Given that the concept of positive welfare in farm animals only began to receive academic attention relatively recently [40], it is interesting to find that it is not a new concept to dairy farmers. Farmers in the focus group consultation defined a good life for dairy cows in terms of opportunities that all related to the same opportunities developed within the scientific literature [14]. A group of self-selecting dairy farmers and welfare scientists broadly agree on what positive welfare means in practice for dairy cattle. There were only two exceptions to this. Farmers did not freely include keeping cows and calves together when defining positive welfare before this opportunity was introduced to them.

There was a new suggestion from farmers that had not been considered by the research team while drafting the framework using the literature. Robotic milking gives cows the opportunity to choose their own milking interval, and this can also enhance comfort by minimising time standing on hard surfaces in the collecting yard and milking parlour, and maximise the cow’s time to express natural behaviours [41]. This opportunity was consequently added to the framework, and is another example of how collaboration with farmers to interpret concepts of welfare can integrate emerging practice.

### 4.6. Support for Farmer-Led Innovation

The on-farm pilot demonstrated that farmers would be willing to make changes necessary to provide some opportunities for their animals with government funding. However, not all opportunities were valued by all farmers. In order for positive welfare opportunities to be delivered in practice, payments alone will not encourage all farmers. Particularly around more controversial or aspirational system changes, understanding the value of change, as well as experiencing the management changes required to facilitate substantive shifts in management is required. Further participatory engagement between the research and farming community is required to employ research and innovation that bridges practical unknown steps and husbandry gaps. The proposed framework is in its infancy and requires a dynamic process of development and refinement as the quality of life literature advances and practical measures for quantifying positive welfare outcomes emerge. Participatory approaches should be central to this process. Using communities of practice to bring farmers together to facilitate discussion, learning and knowledge exchange can support continued innovation across the sector [29,42].

### 4.7. True Cost Accounting

One farmer highlighted that making changes is not only dependent on government payments, but also on public perception and value of milk: if consumers paid more for milk, farmers could afford to make investments to improve opportunities for positive welfare. This calls not only for consumer education on dairy cow welfare, but also for policies to price food in a way that reflects its true production costs, including externalities, known as ‘true cost accounting’ [43].

### 4.8. A Mechanism for Recognition and Reward

The authors would like to acknowledge that the intention of this framework is not that every dairy farm will ultimately be able to achieve every welfare level for every positive welfare opportunity. The purpose is that the framework reflects attainable and aspirational positive welfare opportunities for the spectrum of dairy farmers and systems in operation. Our goal is that there is something for everyone within the framework and that where positive welfare is provided, there is a mechanism for recognition and reward. For example, farmers who are already able to manage their cattle in straw yards, which provides cows with the positive comfort opportunity of choice to lie in any orientation and location on a deep bedded soft and dry surface [44], without compromising cow health.

### 4.9. Future Research Opportunities

In focusing on positive rather than negative aspects of welfare, the proposed good life framework is not a holistic assessment of animal welfare. Further work is needed to evaluate the construct validity of this framework by exploring the relationships between scores and other measures of welfare, such as intended behavioural outcomes, such as play, as well as negative welfare outcomes. Previous research with laying hens found that additional provision of positive welfare opportunities was positively associated with behavioural outcomes, but had no impact on negative welfare outcomes [9].

The total sample size of farmers in the focus groups (n = 10) was small. Therefore, caution must be taken in generalising results to the wider dairy farming community. Although there were only one or two farmers representing each type of system, the dairy farmers who participated represent the full cross section of systems in the UK.

### 4.10. Participatory Policy Development

Dairy farmers self-selected to participate in the focus groups, therefore it is likely that bias is inherent through the participants’ willingness to engage in positive welfare discussions. As such, it should be recognized that the views of this engaged group may differ to those who did not come forward to participate. The views expressed by the focus group are not necessarily representative of the UK dairy farmer population as a whole. However, this work demonstrates the potential for policy to be co-created with farmer focus groups, increasing ownership, relevance and palatability of the end result [29]. We therefore advocate that research scientists, industry and policy makers to use participatory approaches in future policy development [31].

## 5. Conclusions

This study has demonstrated that welfare scientists can utilize farmer focus groups to collaboratively develop a positive welfare framework for dairy cattle. Furthermore, the on-farm pilot phase indicates that dairy farmers appear to be providing additional resources beyond that required by either legislation or certification requirements for which they are currently not receiving recognition or reward for in the market place. Furthermore, all dairy farmers surveyed value some of the positive welfare opportunities presented and stated that they would be willing to implement changes on their farms to achieve these opportunities for dairy cows, as part of a payments by results scheme. Animal welfare is a public good and we are now at a unique opportunity to adapt to consumer/societal demands and progress world leading standards which incorporate existing and emerging positive welfare opportunities to deliver a good life for dairy cattle.

## Figures and Tables

**Table 1:** Percentage of farmers (n = 29) achieving at least one good life resource requirement for each welfare level, expressed as a percentage of the maximum possible achievement of meeting all resource requirements for all welfare levels in that positive welfare opportunity.

Positive Welfare Opportunity	Percentage Welfare +	Percentage Welfare ++	Percentage Welfare +++	Total
Comfort by choice of physical environment	38	9	1	48
Comfort by choice of thermal environment	33	29	7	69
Comfort by choice within environment while minimising harms	41	33	0	74
Comfort by milking choices	21	9	0	30
Pleasure by play and positive social interactions	31	44	0	75
Pleasure by maintenance of the cow-calf bond	23	3	1	27
Confidence by positive experience with stock-keepers, including familiar routines an/processes	43	35	0	78
Confidence by positive learning, resilience and social experiences within the herd	21	40	5	66
Interest by a positively enriched environment	28	7	0	34
Interest by pasture choices	23	18	16	57
Interest by food choices	20	18	0	38
Healthy Life by the stockperson’s knowledge of individual cows’ habits and preferences	72	10	0	82
Healthy life by effective management of day to day health and welfare	44	34	5	83
Healthy Life by positive genetic selection for long-term health and welfare	31	24	21	76

**Table 2:** Positive welfare opportunity or resources most valued by farmers (n = 27).

Positive Welfare Opportunity or Resource	n =	%
Comfort	12	46
Healthy life	11	42
Interest by pasture choices	8	31
All	7	27
Confidence by positive experience with stock- keepers	6	23
Pleasure by play & positive social interactions	3	12
Comfort by choice of physical environment	2	8
Confidence	2	8
Interest by a positively enriched environment	2	8
Comfort by milking choices	2	8
Comfort by choice of thermal environment	1	4
Interest	1	4
Interest by food choices	1	4

**Table 3:** Positive welfare opportunity or resource reported not to be valued by farmers (n = 22).

Positive Welfare Opportunity or Resource	n =	%
Pleasure by maintenance of cow-calf bond	13	59
Interest by a positively enriched environment	4	18
Interest by pasture choices	3	14
Comfort by choice of physical environment: loose housing/covered yards	2	9
Interest by food choices	1	5
Interest	1	5
Pleasure	1	5

**Table 4:** Reasons reported by farmers for not achieving each welfare level for each positive welfare opportunity.

Positive Welfare Opportunity	Capital Investment	Running Costs	Time	Contractual Constraints	Unaware of Benefit	No Welfare Benefit	This Idea Is New	Other
Comfort by physical environment, welfare +	9/12 (75%)	3/12 (25%)	0	0	0	0	0	7/12 (58%)
Comfort by physical environment, welfare ++	4/15 (27%)	10/15 (67%)	7/15 (47%)	1/15 (7%)	1/15 (7%)	2/15 (13%)	0	11/14 (79%)
Comfort by physical environment, welfare +++	9/14 (64%)	3/14 (21%)	0	0	0	2/14 (14%)	0	5/14 (36%)
Interest by pasture choices, welfare +	2/5 (40%)	2/5 (40%)	1/5 (20%)	0	0	1/5 (20%)	0	5/5 (100%)
Interest by pasture choices, welfare ++	2/6 (33%)	2/6 (33%)	1/6 (17%)	1/6 (17%)	0	2/6 (33%)	0	6/6 (100%)
Interest by pasture choices, welfare +++	0	1/7 (14%)	1/7 (14%)	0	1/7 (7%)	1/7 (7%)	0	6/7 (86%)
Pleasure by play and positive social interactions, welfare +	0	0	0	0	1/1 (100%)	0	0	1/1 (100%)
Pleasure by play and positive social interactions, welfare ++	2/5 (40%)	1/5 (20%)	0	1/5 (20%)	0	0	0	3/5 (60%)
Pleasure by play and positive social interactions, welfare +++	2/5 (40%)	1/5 (20%)	0	0	0	0	1/5 (20%)	3/5 (60%)
Pleasure maintenance cow-calf bond +	2/20 (10%)	2/20 (10%)	3/20 (15%)	0	0	8/20 (40%)	0	17/20 (85%)
Pleasure maintenance cow-calf bond ++	2/10 (10%)	2/10 (10%)	3/10 (30%)	1/10 (10%)	1/10 (10%)	6/10 (60%)	0	5/10 (50%)
Pleasure maintenance cow-calf bond +++	3/7 (43%)	3/7 (43%)	2/7 (29%)	2/7 (29%)	0	2/7 (29%)	0	2/7 (29%)

**Table 5:** Other reasons reported by farmers for not achieving each welfare level for each positive welfare opportunity.

	Other Reasons Given
Comfort by physical environment, welfare +	Problems with slurry management; constraints due to TB and increased stocking density; concerns about mastitis risk; does not fit block calving system; health and welfare concerns.
Comfort by physical environment, welfare ++	Disease risk including mastitis; *E. coli* infection risk; poor cleanliness; teat damage; time and monetary costs of cleaning out regularly; do not agree with the concept; health welfare benefits of cows in cubicles outweigh loose housing, supported by quantifiable health key performance indicators (KPIs), e.g., mastitis.
Comfort by physical environment, welfare +++	Issue with vehicle access; rubber mats will become slippery outside; dubious of conclusion in research literature that rubber matting at feed face has measurable benefit.
Interest by pasture choices, welfare +	Issue with ease of access; issue with management due to robotic milking system; not profitable.
Interest by pasture choices, welfare ++	Production concern; practicality of providing shade; weather, ground condition and grass availability concerns; high yielders better managed inside.
Interest by pasture choices, welfare +++	Increased poaching around trees and hedges; decreased cleanliness and increased flies, increased infection risk; impractical and uneconomic; not best for health, welfare or production.
Pleasure by play and positive social interactions, welfare +	Disease risk
Pleasure by play and positive social interactions, welfare ++	Insufficient space; unrealistic for herd size.
Pleasure by play and positive social interactions, welfare +++	Insufficient space; impractical.
Pleasure maintenance cow-calf bond Welfare +	Health issues for dam and calf; safety issues; increased disease including Johne’s disease and mastitis; mastitis milk fed to new born calf likely to result in death; increased stress; risk of mis mothering; impractical due to rate of calving/calving interval; more difficult to teat train; need to get cow into milking routine of robot quickly; increased stress and distress and decreased welfare of cow and calf at separation following bond formation rather than bond never forming.
Pleasure maintenance cow-calf bond Welfare ++	Impractical; uneconomic; increased labour needed; negative welfare for cow and calf; increased disease risk; do not agree with opportunity.
Pleasure maintenance cow-calf bond Welfare +++	Impractical; much reduced milk production; would be more acceptable if use multiple suckled nurse cows; 2 months is not natural weaning; nonsense for dairy herd.

**Table 6:** Likelihood reported by farmers of achieving positive welfare opportunities given government funding.

	If the Full Cost and Time Was Compensated by the Government, How Likely Would You Be to Deliver the Next Resource Tier?			
	Very Likely	Quite Likely	Somewhat Unlikely	Not Likely
Comfort by physical environment, welfare +	3/9 (33%)	1/9 (11%)	2/9 (22%)	3/9 (33%)
Comfort by physical environment, welfare ++	3/11 (27%)	0	3/11 (27%)	5/11 (45%)
Comfort by physical environment, welfare +++	6/7 (86%)	1/7 (14%)	0	0
Interest by pasture choices, welfare +	0	2/5 (40%)	1/5 (20%)	2/5 (40%)
Interest by pasture choices, welfare ++	0	0	0	4/4 (100%)
Interest by pasture choices, welfare +++	0	1/5 (20%)	1/5 (20%)	3/5 (60%)
Pleasure by play and positive social interactions, welfare +	0	0	1/1 (100%)	0
Pleasure by play and positive social interactions, welfare ++	3/4 (75%)	0	0	1/4 (25%)
Pleasure by play and positive social interactions, welfare +++	3/4 (75%)	0	0	1/4 (25%)
Pleasure maintenance cow-calf bond Welfare +	1/16 (6%)	1/16 (6%)	2/16 (13%)	12/16 (75%)
Pleasure maintenance cow-calf bond Welfare ++	0	0	2/9 (22%)	7/9 (78%)
Pleasure maintenance cow-calf bond Welfare +++	1/9 (11%)	0	1/9 (11%)	7/9 (78%)

## Data Availability

The data presented in this study are available in [Table S1].

## Supplementary Materials

The following are available online at https://www.mdpi.com/article/10.3390/ani12192540/s1, Table S1: Development of positive welfare opportunities using scientific evidence, farmer expertise and veterinary assessment. Table S2: Policy development process (Adapted from [29]). References [45,46,47,48,49,50,51,52,53,54,55,56,57,58,59,60,61,62,63,64,65,66,67,68,69,70,71,72,73,74,75,76,77,78,79,80,81,82,83,84,85,86,87,88,89,90,91,92,93,94,95,96,97,98,99,100,101,102,103,104,105,106,107,108,109,110,111,112,113,114,115,116,117,118,119,120,121,122,123,124,125,126,127,128,129,130,131,132,133,134,135,136,137,138,139,140,141,142,143,144,145,146,147,148,149,150,151,152,153,154,155,156,157,158,159,160,161,162,163,164,165,166,167,168,169,170,171,172,173,174,175,176,177,178,179,180,181,182,183,184,185,186,187,188,189,190,191,192,193,194,195,196,197,198,199,200,201,202,203,204,205,206,207,208,209,210,211,212] are cited in the supplementary materials.
