# Supplementary material for: A “Good Life” for Dairy Cattle: Developing and Piloting a Framework for Assessing Positive Welfare Opportunities Based on Scientific Evidence and Farmer Expertise
Source: Animals (Basel). 2022 Sep 22;12(19):2540. doi: 10.3390/ani12192540 (PMC9559654; doi:10.3390/ani12192540)

Supplementary Tables

Table S1 Development of positive welfare opportunities using scientific evidence, farmer expertise and veterinary assessment

**Positive Welfare Opportunity for Comfort** By choice of physical environment<br>*Cows should be able to exercise individual preferences for their physical comfort at all times*
**Initial proposal based on review of scientific evidence**<br><br>**Law** Where any animals are kept in a building, they shall be kept on, or have access at all times to, a lying area, which either has well-maintained dry bedding or is well drained.<br>**Code** Where there are slats, part of the accommodation should be a solid-floor area with straw or some other suitable bedding material. There should be enough space for all the animals to lie in comfort at the same time. Cubicles should have enough bedding to keep the cows comfortable.<br>**Welfare +** : Where any animals are kept solely or partly in a building they should be loose-housed [44–46], or at minimum have free access to an unrestricted/ undefined lying area that allows animals the choice to lie in any orientation and to switch sides easily [47]. Deep (i.e. compressible) and dry bedding in indoor lying areas [48–51]. Choice to avoid hard surfaces outdoors (for example to walk on verge rather than road).<br>**Welfare ++** : As above plus a choice of different lying areas and substrates at some times of day [45,51].<br>**Welfare + + +** : As above plus a choice of different lying areas and substrates at all times [45,51].<br><br>**Validity**<br>Dairy cows are highly motivated to lie down for up to 14 hours per day. Cows will work (press repeatedly on a panel) to increase lying times [52]. Lying is a high priority behaviour, even higher than eating and social contact when opportunities to perform these behaviours are restricted [53]. Lack of lying and sleep has an impact on welfare and production. When cows had access only to stalls freshly bedded with kiln-dried sawdust, lying times averaged almost 14 hours per day [50,54]. In contrast, when cows had access only to stalls with wet bedding, lying time decreased to <9 hours per day [54]. Cows ruminate and produce saliva optimally while lying down, reducing ruminal acidosis. A lying cow has an increased blood diffusion through the udder (around 5 L / min) compared with a standing animal (around 3L / min). Lying comfort is fundamental for optimal vitality, udder function and milk production. Repeated and sustained standing and walking on hard surfaces is a significant risk factor for lameness. Competition for a comfortable resting place can trigger social conflicts between cows.<br>**Feasibility**<br>When assessed as part of the whole good life framework pilot, it took approximately 5 minutes to assess comfort by choice of physical environment. It is possible that this time could be reduced with a more detailed protocol, training and experience. This measure is relevant and applicable to all dairy groups (cows, heifers, calves and bull(s)).<br>Between or within assessor variation has not been formally tested on farm. However, with well-defined protocols, adequate guidance with practical examples and training, assessment of the choice and quality of physical resources animals have access has potential as a positive welfare indicator. Further research should focus on further developing and validating this measure in collaboration with farmers, vets, advisors and farm assurance assessors.

As this opportunity requires a grading of resources which has previously taken 5 minutes to carry out, it is practical for both farmers and farm assurance assessors to carry out. However, free-standing cubicle housing has been the most common method of housing dairy cattle in the UK since the 1960s. Changing back to loose housing from cubicles requires a farmer to perceive benefits that outweigh the perceived costs (i.e. mastitis control), a change in management strategy and potentially a very large investment of capital. As farmers believe mastitis to be one of the most expensive diseases where most farmers have changed their whole management system accordingly and put their greatest efforts into mastitis control [55] it may be very difficult to encourage adopting the management changes required to meet this resource tier. This opportunity as a potential measure of positive welfare is more palatable to new or existing loose housed systems, as a means of recognition and motivation for further positive welfare improvement i.e. giving cows choice of several lying substrates and monitoring the changes overtime.

Relevant focus group suggestions for defining "What is a good life for your cows?"

Percentage of herd lying down

Lying comfort – space for ease of sitting and standing

Deep litter bedding or cubicles?

Undisturbed lying area

Comfortable bedding and flooring

Comfy sand beds to lie in as long as cows want

Summary of focus group discussion

Comfort was identified in meeting one as a means of defining positive welfare. In practice, this was articulated as providing comfortable, deep bedded, undisturbed, spacious and easily accessible areas for cows to lie in for as long as they want to. In meeting two, the focus group articulated that cow comfort is one of the most important opportunities to provide cows with positive welfare. Cow comfort was a major priority for cow welfare, which farmers felt very proud to deliver. Although the group agreed with the literature that loose housing offered the choice to exercise individual preferences by having access to an unrestricted, undefined lying area, there was extensive discussion about the fact that in order to motivate farmers to engage with a framework like this in practice, each potential opportunity should in principle be accessible to all. This coupled with the fact that the majority of dairy cows in the UK are housed in cubicles, and it was felt that the significant difference in comfort and degree of choice in cubicle housing delivered on the ground should be recognised at the Welfare + level. It was also acknowledged that in order to warrant Welfare + in a predominantly cubicle based system; loose housing was an imperative requirement for cow comfort and choice for at least two weeks before and after calving. In addition, the focus group felt that in order to warrant Welfare ++, it was the farmers and stock keepers responsibility to monitor cow comfort using either cameras in the housing, activity meter data, behavioural observations and/or welfare outcome scoring. This would equip stock keepers with a tool to adjust resources or management to improve cow comfort where necessary. This requirement was therefore added to Welfare ++ of the healthy life by effective management of day to day health and welfare opportunity as a monitoring tool which could be used to measure the impact of cow comfort and a number of other resources proposed in other tiers of the framework. Finally, farmers emphasised the importance of the provision of rubber matting for underfoot comfort at prominent standing places while cows were housed, in order to provide choice of comfort in the standing environment (see Welfare +++).

Amended protocol after consideration by farmers in focus groups

Welfare + : Where any animals are kept solely or partly in a cubicle buildings, they must use either deep bedded sand or a similar soft, compressible substrate of at least 6 inches in depth, maintained clean and dry. Cubicles must be of an appropriate design and size for the herd, and should ideally allow cows to put their feet out to the side. There must be 10% more cubicles than cows to allow some choice of where to lie and who to lie next to. Farm provides loose housing as per welfare ++ for a period of at least two weeks before and after calving.
Welfare ++ : Where any animals are kept solely of partly in a building they must be loose-housed having free access to an unrestricted/undefined lying area that allows all animals to lay in any orientation and choose where to lie and who to lie next to. The bedding is at least 6 inches of compressible dry and clean bedding. There is at least 10% more lying space than the space the cows take up.
Welfare +++: As Welfare ++, plus the farm provides rubbing matting at prominent standing places e.g. feed face, collecting yard and in the milking parlour.

Final data collection protocol after review by veterinary surgeons

Welfare + If cows are kept solely or partly in cubicle buildings, do they use either deep bedded sand or a similarly soft compressible substrate?
Welfare + Is the bedding at least 6 inches, dry and clean?
Welfare + Are cubicles a suitable design and size for the herd?
Welfare + Do the cubicles allow cows to put their feet out to the side?
Welfare + Are there at least 10 more cubicles than cows to allow choice of where and who to lie next to?
Welfare + Does the farm provide loose housing as per Welfare +++ for a period of at least 2 weeks before and after calving?
Welfare ++ Does the loose housing provide unrestricted/ undefined lying area that allows all animals to lay in any orientation and choose where to lie and who to lie next to?
Welfare ++ Is there at least 6 inches of compressible, dry and clean bedding
Welfare ++ Is there at least 10 more lying space than the space the cows take up?
Welfare +++ Does the farm provide rubber matting at prominent standing places i.e. feed face, collecting yard and parlour?

Positive Welfare Opportunity Comfort By choice of thermal environment
Cows should be able to exercise individual preferences for their thermal comfort at all times

Initial proposal based on review of scientific evidence
Law: Temperature kept within limits that are not harmful. Where any animal is kept in a building it should have access at all times to, a lying area, which either has well maintained dry bedding or is well drained. Animals not kept in buildings shall, where necessary and possible, be given protection from adverse weather conditions.
Code: For grazing animals, shelter or natural shade from trees or hedges is important in summer. Where appropriate, roofs should be insulated to reduce solar penetration.
Welfare + : Housed animals of all ages have a choice of temperatures within their indoor environment (for example nearer to or further from open or ventilated areas) and they can always avoid thermal extremes.
Welfare ++ : As above, plus cows can choose to be indoors or outdoors for at least part of the day/night and shelter availability is sufficient to mitigate for effects of outdoor weather [56], e.g. sufficient hedgerow/ trees for all animals to stand or lie in the shade at the same time.
Welfare +++ : As above, plus a choice of temperatures and shade intensities within their outdoor environment, e.g. choice of shelters/ shade areas with varying protection from solar radiation [57,58].

Validity

When cows were kept outside in winter weather conditions without shelter, they spent less time lying down, had higher cortisol, glucocorticoid, T4 and NEFA concentrations [56]. They were also more likely to spend time in lying and standing postures that reduced the amount of surface area exposed to rain and wind [56]. Shade use increases with higher levels of solar protection [58]. Cows were more likely to use shade structures when ambient solar radiation levels were highest within a day and cows with more protection from solar radiation had lower minimum body temperature [58]. In addition, aggressive interactions in the shade, time around the water trough, mean body temperature, and respiration rate increased with environmental heat [57]. Weather conditions influence the behavioural and physiological responses of cows, and these changes are more pronounced when less or no shade and shelter is available. Indoor housing, and outdoor shelter and shade give cows the opportunity to regulate their own thermal temperature and maximise performance of natural foraging, resting and social behaviours.

Feasibility

The comfort by choice of thermal environment measure is relevant and applicable to all dairy groups (cows, heifers, calves and bull(s)).The reliability and feasibility of using this potential measure as an on farm indicator of positive welfare has not been formally assessed. However, when piloted as part of the whole good life resource tiers framework, each measure took approximately 5 minutes to assess. This is not prohibitively time consuming for farmers, vets, advisors or farm assurance assessors.

The repeatability of using this measure as a positive welfare indicator over time has not been formally assessed and should be part of future research. Devising protocols and adequate guidance with practical examples for training and standardisation purposes should be developed in conjunction with farmers and farm assurance assessors, including an assessment of both between and within assessor reliability over time.

This measure has yet to be applied on farm as an indicator of positive welfare but as part of a resource tiers framework pilot study, the assessment of each resource took approximately 5 minutes. It therefore appears feasible to carry out an assessment of comfort by choice of thermal environment by farmers, vets, advisors and farm assurance assessors. The choice of access to both an indoor and outdoor environment is a fundamental differentiation between higher welfare farming systems, in particular, free range laying hens, pigs and dairy. In 2015, a group of dairy farmers create their own independent free-range dairying brand [12]. In 2016, Waitrose became the first supermarket to commit to assuring all dairy cows in their supply chain have at least 100 days access to pasture [59]. Research shows that access to pasture needs to be in conjunction with adequate shade and shelter, otherwise animals cannot utilise the maximum benefit the choice of the outdoor environment, particularly during weather extremes. Providing trees and hedgerows around pasture for animals to roam forage and browse could facilitate both higher welfare and environmental sustainability outcomes.

Relevant focus group suggestions for defining "What is a good life for your cows?"

Not exposed to temperature stress

Give cows different spaces so they can chose to stand in the yard if it is sunny or go into the building if it is raining

Summary of focus group discussion

Thermal comfort was identified in meeting one as a positive welfare attribute. In their own words, farmers defined thermal comfort as cows not being exposed to temperature stress, and giving cows different spaces so they can chose to stand in the yard if it is sunny or go into the building if it is raining. In meeting two, the focus group agreed with the principle of choice of thermal comfort within the indoor and outdoor environment, and the resource proposed to deliver increasing levels of thermal choice. One addition was proposed - an example of a resource that could deliver this opportunity in the outdoor environment where growing natural cover was not possible, or where trees and hedgerows had been planted but were not yet mature enough to provide the opportunity of thermal comfort when weather was too hot/wet. The example given by one farmer was portable shade havens. This example was added but the wording of this opportunity did not change significantly.

Amended protocol after consideration by farmers in focus groups

Welfare + Housed cows have a choice of temperatures within their indoor environment (for example nearer to or further away from open or ventilated areas) and can always avoid thermal extremes (for example; fans and ventilation for heat, no excessive drafts in cold weather).

Welfare + + As above, plus cows have some access to the outdoors in the winter and summer (i.e. loafing area, paddock or grazing). Shelter is sufficient for all cows to use at the same time and mitigate the effects of outdoor weather, particularly rain and sun e.g. sufficient hedgerow, trees and/or portable shade haven shelters.

Welfare + + + As above, plus a choice of temperatures and shade intensities within their outdoor environment, e.g. hedgerows, trees and portable shade haven shelters which create areas with varying protection from solar radiation.

Final data collection protocol after review from veterinary surgeons

Welfare + Do housed cows have a choice of temperatures within their indoor environment? (Nearer to or further from open or ventilated areas).

Welfare + Can housed cows always avoid thermal extremes? (For example fans and ventilation for heat, no excessive drafts in cold weather)

Welfare ++ Do cows have some access to the outdoors in the winter? (Loafing area, paddock or grazing).

Welfare ++ Do cows have some access to the outdoors in the summer? (Loafing area, paddock or grazing)

Welfare ++ Is shelter sufficient for all cows to use at the same time and mitigate the effects of outdoor weather?

Welfare +++ Is there a choice of temperatures/shade intensities i.e. hedgerows and trees or portable shade havens with varying protection from solar radiation?

Positive Welfare Opportunity Comfort By choice within environment while minimising harms

Cows should be able to exercise individual preferences within their environment whilst minimising associated harms

Initial proposal based on review of scientific evidence

Law: Accommodation and fittings for securing animals shall be constructed and maintained so that there are no sharp edges or protrusions likely to cause injury to them.

Animals not kept in buildings shall, where necessary and possible, be given protection from adverse weather conditions, predators and risks to their health.

Code The floor should not slope too steeply – no more than about 10%. All concrete yards and passageways should be kept in good condition, not too smooth or rough and slurry should not be allowed to build up. Gaps between slats should not be wide enough to cause foot injuries.

Welfare + Standing/ lying/ loafing areas, feeder spaces, watering points and walkways/ passageways are designed to enable cows to avoid harm from other cows, stock persons and from their physical environment

(such as pushing along, bullying, crowding, bottle-necks) [60]. Lighting within buildings should be appropriate to enable animals to exercise the choices described in other sections, to avoid harms and to sleep comfortably.

Welfare ++ : As above, plus a choice of standing/ lying/ loafing areas, feeder spaces, watering points and walkways/ passageways, to reduce negative social interactions. Cows have opportunities to exercise preferences within familiar routines, such as laterality in cubicles, loafing areas, parlours and on tracks [47].

Welfare +++: As above, plus a policy for monitoring the animals' use of resources/ opportunities for positive welfare in order to maintain or enhance these, and a policy for monitoring and acting on incidence of injuries and negative social interactions between cows. The health plan should demonstrate how the provision of opportunities for positive welfare is balanced with prevention of harms; for example, how opportunities for play and social interaction are balanced with minor injury, or opportunities for food choice are balanced with appropriate nutrition.

Validity

The relationship between positive stockmanship, animal welfare and productivity has been thoroughly documented. Studies consistently indicate a close link between positive attitudes towards livestock, positive behaviours by the stock people, and the stress responses and productivity of animals [60]. Ensuring easy access to feed, water, and resting space for all cows at all times is at the centre of good management practice. Social stressors (rank, regrouping, competition over resources, stocking density, displacement) have an impact on immunity, health and welfare. For example, when animals were ranked based on feed bunk displacement, dominant animals were far more likely to be diagnosed with metritis than subordinate animals [61]. Lying bouts of more than 80 minutes without the opportunity to switch sides/lie in any chosen direction is uncomfortable for dairy cows [47]. Lack of comfortable lying has a negative impact on sleep, productivity and welfare, and can further lead to injuries such as hock swellings and lesions [62].

Feasibility

As part of an initial pilot study, it took approximately 5 minutes to assess Comfort by choice within the environment while minimising harms. However, only a small number of farms were visited (17). A much larger sample, in conjunction with farmers is required to fully test feasibility. As the behavioural outcome of this potential positive welfare indicator is positive interactions between cows and stock people as well as minimising environment harms caused by injury, it is logical to develop and use behavioural and animal-based outcomes to measure this. Welfare outcome assessment already developed [63] is applicable to measure injury caused by the environment. A next step would be to identify existing behavioural outcome measures or develop these in conjunction with stock people to verify positive social interactions between cows and stock people.

The repeatability of assessing choice within the environment while minimising harm has not yet been tested on farm. Further research should focus on further developing and validating behavioural outcomes in collaboration with farmers, vets, advisors and farm assurance assessors.

With the inclusion of welfare outcomes and the development of positive behavioural outcomes, the choice within the environment while minimising harm has potential to be used as a positive welfare indicator by farmers, vets, advisors and farm assurance assessors - to identify areas for continuous improvement, benchmark within and between farms, as well as identifying differences in and between farming systems.

Relevant focus group suggestions for defining "What is a good life for your cows?"

Comfortable housing

Understock cows

**Summary of focus group discussion**<br>In meeting one farmers defined positive welfare as providing housing that is more comfortable and understocking cows to provide more space in the indoor environment. In meeting two the focus group agreed with the principle of comfort by choice within the environment while minimising harm and the proposed resource choices required to deliver increasing levels of this opportunity in practice. Although the group agreed that monitoring the use of the existing resources to achieve this opportunity was a valuable positive welfare farm policy, as with monitoring physical comfort, it was deemed clearer and more efficient to have a monitoring requirement for all relevant resources within the opportunity for healthy life by effective management of day-to-day health and welfare. The focus group suggested one addition to the wording of Welfare ++ level, for the farm environment to be assessed as calm and quiet. Due to removing the monitoring system in favour of putting this in the healthy life opportunity, comfort by choice within environment while minimising harm progressed with two higher welfare levels (**Welfare +** and **Welfare ++**)
**Amended protocol after consideration by farmers in focus groups**<br>**Welfare +** Standing/ lying/ loafing areas, feeder spaces, watering points and walkways/ passageways are designed to enable cows to avoid harm from other cows, stock persons and their housing environment (no forced movements, pushing, bullying, crowding, or bottle-necks). Lighting within buildings is appropriate to enable animals to avoid harm, exercise, relax and sleep comfortably.<br>**Welfare ++** As above, plus a choice of different types/areas for standing/ lying/loafing, feeder, watering points, walkways and passageways, to enable cows to exercise individual preferences of the resources they use within familiar routines. The farm environment is calm and quiet.
**Final data collection protocol after review from veterinary surgeons**<br>**Welfare +** Are standing, lying and loafing areas, feeder spaces, watering points, walkways and passageways designed to enable cows to avoid harm from other cows, stock people and the housing environment? (no forced movements, pushing, bullying, crowding, or bottle-necks).<br>**Welfare +** Is lighting within buildings appropriate to enable animals to avoid harm, exercise, relax and sleep comfortably?<br>**Welfare ++** Is there a choice of different types/areas for standing, lying, loafing, feeding, walk and passageways to enable cows to exercise individual preferences?<br>**Welfare ++** Is the farm environment calm and quiet?
**Positive Welfare Opportunity Comfort** By milking choices<br>Cows are able to exercise individual preferences for the frequency and duration of milking
**Initial proposal based on review of scientific evidence**<br>No specific proposal on milking choice
**Relevant focus group suggestions for defining "What is a good life for your cows?"**<br>Minimise time in collecting yard and milking – allow maximum time for everything else<br>Robotic parlour – cows choose milking interval
**Summary of focus group discussion**<br>Given robotic milking gives cows the opportunity to choose their own milking interval, and this can also enhance comfort by minimising time standing on hard surfaces in the collecting yard and milking parlour, and maximising the cow's daily budget of time to express natural behaviours, in meeting two, the focus group proposed an additional opportunity for comfort by milking choices.

Amended protocol after consideration by farmers in focus groups
Welfare + Time spent standing in collecting yard waiting to be milked is dramatically reduced by bringing cows around to go straight into the milking parlour in small groups according to their need of milking, or letting cows come into the collecting yard in their own time.
Welfare ++ Cows have the opportunity to choose their own milking interval as the farm uses a robotic milker.

Final data collection protocol after review from veterinary surgeons
Welfare + Are cows left to come into the collecting yard in their own time or brought around in small groups (i.e. 20 cows at a time) without the use of human physical force or a backing gate?
Welfare ++ Do cows have the opportunity to choose their own milking interval because the farm uses a robotic milker?

Positive Welfare Opportunity Pleasure By play and positive social interactions
Cows should be able to experience positive social interactions and have space and time for play and affiliative behaviours

Initial protocol based on scientific evidence
Law The law does not detail requirements about play and positive social interactions.
Code The codes do not detail requirements about play and positive social interactions.
Welfare + Social and physical environment enables social sniffing, licking and allo-grooming at all times during all life stages [64–66].
Welfare ++ As above plus space, time and layout of housing/ pasture allow **some opportunities** for play including locomotor (running) and social (chasing/ rough-and-tumble) play behaviour [18,20,67] during all life stages.
Welfare +++ As above, but opportunities for play including locomotor and social play behaviour are available **at all times** during all life stages.

Play behaviour
Play behaviour includes functional elements, such as fleeing, play fighting (solicitation, horning and pushing), sexual, or predatory behaviour as well as specific play behaviours. In play, the functional elements are exaggerated, repeated, more variable, and without the corresponding consummatory act. Functions of play include training of the skeletal muscles, self-assessment of physical and social abilities and training of flexible kinematics and emotional responses to unexpected events. Play is a highly rewarding activity that aids cohesion of the group. Animals actively seek out play partners and solicit play behaviour. Play fighting aids social contact and serves to avoid social conflicts. Dairy calves start to perform solitarily locomotor play (bucking, trotting, and cantering) at a few days old and social play with a partner from a few weeks old. For example, social play fighting was frequently seen in infants in a semi-wild herd, but less in adults apart from calves soliciting play with their mother. Young animals can spend up to 10% of their total active time and energy on play.
Play elicits a positive emotional state(s). Animals positively anticipate opportunities to play. Lambs anticipate play as a forthcoming reward. Thwarting of play leads to a rebound when favourable conditions of space, a play partner or enrichment re-occurs in dairy calves. Similarly, lambs express play behaviours when moved from their pens to a larger area containing enrichment objects. However, play or the anticipation of play has yet to be developed for application on farm as a positive welfare indicator.
Validity
Elevated cortisol levels have been correlated with decreased play. In contrast, play may induce anti-stress effects through oxytocin release, thereby lowering cortisol levels. Animals kept in restricted environments show both

physiological (higher adrenal response, reduced immune fitness) as well as increased locomotor (bucking, trotting and cantering), social play (rubbing and butting heads) and social licking behaviours when moved into a more spacious area. Food availability and thermal comfort are prerequisites for the expression of play. Equally, if an animal is injured or ill, play is found to be absent. Therefore, despite being highly motivated to play, animals only perform play behaviours when the environment is sufficiently enriched and their primary needs are met. Play is not performed under stressful conditions or a stressed state. The prevalence of play could therefore indicate both the absent of negative welfare and presence of positive welfare.

Feasibility

The feasibility of capturing play behaviour as an indicator of positive welfare in a commercial dairy herd has yet to be investigated. Locomotor play is more common very early (from a few days old), whereas social play is observed from the second week of life onwards. Locomotor play peaks at 2 weeks of age, social play increases from 2 weeks up to 6 months of age. Social play takes place primarily between partners of the same age, although calves will solicit play with older and younger partners. Social play between the mother and calf is initiated by the calf. From 2-3 months, social play mainly occurs between partners of the opposite sex. Activity among male calves elevates between 4 and 10 months of age, and by 30 months of age play fighting between males is 60-times more frequent than female-female and 30-times more frequent than female-male pairs. Observations in a semi-wild cattle herd suggest social play activity peaks just before midday and early evening rest. Measuring the environmental prerequisites for play across farming systems, and establishing the time periods when play is likely to occur will increase the feasibility and practical sampling methods. More recent research with other species suggests that the propensity to play can vary significantly between individuals, and this may relate to differences in personality which impacts on future behaviours, such as anxiety and exploration. Investigating individual differences in the propensity of cows to play could help further understand what play behaviour indicates about quality of life. Manual capture of play behaviours on farm is only practical in a commercial setting on farm if carried out by the caregiver(s). In time, recording equipment and software that automatically detects and codes play behaviours could increase feasibility and standardisation if not prohibitively expensive to apply.

As play has not yet been applied on farm repeatability within or between assessors has not been tested. However, clearly defined behaviour descriptors and video recordings could be used to train caregivers and validate play behaviours. An automatic system using algorithms to identify and record play behaviour could save time and validate caregiver observations. There may however be more intrinsic value for caregivers directly measuring the environmental prerequisites of play and observing their animals for play behaviours, in terms of both enhancing their own welfare and motivating changes to promote further play opportunities to facilitate positive welfare.

The presence of play behaviours could indicate to the caregiver that their animal's primary needs are met, the environment is sufficiently enriched and the animals are not in a stressed state. Play can therefore be seen as a signal of both the absence of negative welfare and the presence of positive welfare. Play behaviours in calves and young animals is well defined but more work needs to be done to understand opportunities for play in older animals. As play is not performed as frequently as maintenance behaviours, on farm development needs to identify under what environments and times of day play behaviours are likely to occur, to develop feasible, practical sampling approaches. Due to the propensity of play behaviours, manual capture will only be practical by caregivers. If caregivers want to promote play behaviours which bring their animals pleasure, developing a system to identify, monitor and facilitate play can provide a tool to communicate added value to their customers

and stakeholders. The value of observing animals for positive behaviours like play can act as a positive feedback reward to encourage more farmer led promotion of pleasurable behaviours on farm.

Relevant focus group suggestions for defining "What is a good life for your cows?"

Group housed calves as they play more than when on their own

Space for calves to play

Allow cows to play with their environment

Summary of focus group discussion

In meeting one, farmers defined positive welfare as cows being able to groom each other, for calves to have other calves to play with, as well as resources within the environment for cows to play with. In meeting two the focus group agreed with the principle that pleasure can be achieve through the opportunity for play and positive social interactions within the herd. The farmers expressed that it was particularly important to rear calves in a social group from the outset, or at least pairs then later added to a bigger group which they follow through to bulling to facilitate play and affiliative behaviours (Welfare +). One additional clarification was added to Welfare +++ with regards to the practicalities of not being able to provide this opportunity while cows were calving, milking or hospitalised.

Amended protocol after consideration by farmers in focus groups

Welfare + The social and physical environment enables social sniffing, licking and allo grooming at all times.

Welfare ++ As above, plus cows have space, time and layout of housing/pasture which allows some opportunities for play including locomotor and social play behaviour.

Welfare +++ Cows have the opportunity for locomotor and social play available at all times (Excluding calving, milking or hospitalisation).

Final data collection protocol after review from veterinary surgeons

Welfare + Does the social and physical environment enable social sniffing, licking and allo grooming at all times?

Welfare ++ Do the cows have space, time and layout of housing/pasture which allows some opportunities for play including locomotor and social play behaviour?

Welfare +++ Do cows have the opportunity for locomotor and social play available **at all times?** (excluding calving, milking or hospitalisation)

Positive Welfare Opportunity Pleasure By maintenance of the cow-calf bond

Cows should be able to experience maternal bonding and calves should be able to bond with and learn from their dam

Initial protocol based on scientific evidence

Law The law does not detail requirements about cow-calf bond.

Code Ideally, calves should be left with their dam for at least 12 and preferably 24 hours after birth. It is recommended that the calf should continue to receive colostrum from its mother for the first three days of life. Removing the calf earlier than 12-24 hours after birth should only be done for disease control purposes, under the advice of a veterinary surgeon and the protocol should be recorded in the health and welfare plan.

Welfare+: Calves are kept with a lactating dam (may be multiple suckling) for at least several days [67,68], and after separation calves are kept within sight of the dam [69–71] although [72,73] mixed findings if separated after 24hr but less than a week. Calves are separated onto a teat rather than a bucket [67] and warm water is substituted for milk in the milk-feeding apparatus for the first 2 days after weaning off milk [74] and/or progressive dilution of milk with water [75].

Welfare++: As above, but calves are gradually weaned using a two-step method (suckling prevention then separation) [76,77] or a contact weaning method (e.g. anti-suckling devices then fence-line contact then visual contact) [67,78].

Welfare+++: Calves are kept with their own dam until natural weaning [69,79–81].

Maintenance of the cow calf bond

When calves are allowed to stay with the cow a strong bond develops and the bond typically weakens as the calf becomes less reliant on milk. At weaning, the young mammal must make the transition from reliance on the dam and the milk she provides to more social and nutritional independence. In the wild (or under naturalistic conditions) nursing frequency and milk transfer gradually decline after the first few weeks of life as calves begin to graze and ruminate. However, calves typically continue to receive some milk from the dam until 6 to 9 months of age [82].

In contrast, in a commercial system the dam is typically separated from the calf between a few hours and 24 hours of birth, and fed milk by bucket or bottle until weaned from milk to solid food weeks later. Dairy calves are typically separated soon after birth as a number of studies have suggested that calf and dam response to separation, namely vocalisation, increases with calf age [83]. Calves show a pronounced behavioural response when both separated from the cow, and later weaned from milk. Vocalisations are a natural adaptation specifically designed to signal a continued need for milk and maternal care and modify the behaviour of the dam. Cows also show a pronounced behavioural response, through vocalising, agitation (stopping, stepping back, stamping and kicking) and increased cortisol level which remain marked for 2 days after separation, with some cows still reacting for up to 7 days post weaning [84]. In commercial dairy systems weaning typical involves separation from the dam and a change in diet from milk to solid food, but can also involve a change in physical environment (different housing, drinkers, feeders, smells and sounds) and social environment i.e. mixing with unfamiliar calves. These changes can cause accumulative stress and disentangling their impact can be a challenge. However, research suggests that the strength of the behaviour response at weaning increases with the number of stressors imposed at once and in line with the calves need for the various aspects of maternal care [85].

Validity

Neurochemicals known to play a role in inducing positive affective states are involved in pair bond attachment, sucking and satiety, and different neurochemicals play a role in different aspects of maternal care. For example, calves release oxytocin during milk intake [86], but the presence of oxytocin only increases when calves are nursing from the cow and not when drinking milk from a bucket which suggests the cow and/or the opportunity to suckle is important for oxytocin release. Oxytocin is also involved in vocalisations heard at separation. Oxytocin decreases as separation induced calling increases [87]. Although yet to be studied in farm animals, vasopressin appears to function in conjunction with oxytocin at the early stages of attachment, as well as having a role in promoting social behaviour in later life [88]. Furthermore, cow milk contains the tetra peptide, alpha-Lactophorin, which is structurally similar to endogenous opioids and binds to opioid receptors [89]. This has led to one theory that young calves develop an addiction to milk and the phases of attachment are similar to the withdrawal symptoms of opiate addiction [88]. The neurochemical cholecystokinin is also reduced when calves do not have the opportunity for (non-nutritive) sucking [90]. As well as the physiological and behavioural functions, we are starting to understand more about the significance of the cow-calf bond for cognition and social learning. For example, a recent study demonstrates that separation from the dam evokes a pessimistic judgement bias indicative of a negative emotional state of low mood similar to that experienced by calves in pain in the

hours after hot-iron dehorning [91]. The authors suggest that separation which brings about social loss induces a high intensity negative affect more similar to anxiety than depression. This study provides the first evidence of a cognitive response to separation from the dam in any farm animal species. We are also starting to understand how the quality of the dam calf interaction influences the cow's social skills and cohesion within the herd. Social learning is a vital part of understanding social cues to establish and maintain affiliative relations within the herd. When calves are allowed to stay with their mothers, they initiate play. Performing play behaviours are particularly important in learning social skills required for positive social interactions within herd in later life. For example, heifers that had been allowed to suckle their dam tended to show more submissive and non-confrontational postures than heifers that were reared on the automatic milk feeder without contact with adult cows [79]. More recent research has demonstrated the importance of the dam for the calves' cognitive abilities. For example, dam reared calves at 10 weeks of age explored and tasted novel feed sooner and ate more of it, compared to calves individually reared [92]. This has obvious production benefits. Furthermore, dam rearing improves the calf's ability to carry out 'reverse learning' a marker of cognitive ability [93] which has implications for a cows resilience to adapt to unpredictable events in their environment. Maternal deprivation reduces an animal's capacity to cope with normal social interactions within the herd [94]. It has been found that calves reared without contact with their dam show a decline in learning and social skills [75,95] and an increased responsiveness to stress [96]. Dam reared cows show more exploratory behaviour [97] and are more active [80]. This has also been shows in dam reared calves and is attributed to a higher social motivation to join the herd. Re-introducing recently calved heifer to the milking herd can be particularly stressful [97]. Mothering a calf influences the behaviour of other cows in the herd. Further research following the long term effects of dam rearing on other behaviours (for example oestrus behaviour) are needed, as well as understanding the effect of maternal behaviour on social learning, cognition, coping with stressors and health of cows over several generations (Johnson et al., 2016). Maternal behaviours by the dam, nursing, and cow-calf bonding which include affiliative behaviours such as licking, rubbing and staying close for security are important natural behaviours [98]. The presence of a dam has a strong effect on the development of calves' oral behaviours and drinking milk from a bucket does not satisfy the desire to suckle [99]. Cows that suckled their dams as calves licked and nursed their own calves longer than conventionally reared cows [100]. Calves suckled on their dam compared to calves fed artificial from birth tended to spend less time on non-nutritive oral activities after weaning (at 42 days of age) which included licking or sucking another calf or an object, nibbling at an object, and tongue-rolling [84]. The dam is also an important social feeding model for the calf. Calves learn what to eat and what to avoid by following their mothers choices [101]. Frequency of solid feed consumption was three times higher in lambs that were left in continued social contract with the ewe (but prevented from nursing), in comparison with lambs separated from the ewe [102]. Thomas et al., 2001 [103] found that calves call more when hungry than well fed, as vocalisations after separation from the dam were found to peak at 18 hours. It has also been found that calves given double the amount of colostrum normally provided showed almost no vocalisation response to separation from the dam. Calves show less behavioural response to dilution of their milk ration (warm water) so long as this change in diet is not combined with other known stressors such as separation from the mother [104]. Furthermore, Haley et al., 2005 [77] found that calves that were prevented to suckle but allowed full social contact with their mother vocalised much less when later separated from the cow, compared to calves that were allowed to suckle right up to the time of separation. Therefore, encouraging calves to gain nutritional dependence before separation can reduce the vocalised stress response (for example, fence line weaning [105]). Increased feed intake and weight gain is found in animals weaned at older ages [106]. Calves can choose the frequency of meals and meal sizes that fit their physical needs when kept with the dam (or as part of a foster care system). As a result,

the weight gain of free suckling calves can be higher than that of calves reared without the dam [107,108]. However, where calves are separated at 8-12 weeks (compared to natural weaning at 8-12 months of age) and this is accompanied by an abrupt shift from milk to solid food, a period of low weight gains post weaning results [108,109]. Increasing solid feed intake and nutritional independence from the dam before weaning and separation takes place is paramount in reducing stress and the impact on continued calf development and production [110]. Half day or night contact systems have shown promising results recently. This system can give the calf an opportunity to form a bond and perform bonding behaviours, (interestingly, although suckle induces positive affective states it is not required to form the bond) and become nutritionally more independent from the dam before separation [36]. Authors suggest an udder net maybe a viable way of allowing cow calf contact without suckling and the associated loss of milk production. Zebu cattle (B. indicus) and cross breeds of Zebu nursing their calf shortly before and after each milking, are reported to yield a similar [111] or higher [112] amount of saleable milk compared to cows that are not suckles by their calves. However, the opposite affect is observed in European dairy cows (B. taurus) where the amount of saleable milk is reported to reduce by 7-12kg in restricted suckling systems and up to 20kg per day in free contact systems [113,114]. However, this is because calves drink more than they are given in conventional dairy systems and milk ejection is sometimes impaired by suckling cows. Nursing cows release less oxytocin during milking compared to non-nursing cows which may suggest an aversion to being milked or because the cow is keeping her milk for the calf [113]. One solution is to have the calf present at milking (without suckling) as this has been shown to improve milk let down [115]. Although this would be difficult in present modern dairy set ups, it has been reported anecdotally that some farmers have solved this in practice by putting the calves in a pen in front of the parlour which is visible to the cow [85]. One major health and production concern for the dairy industry is mastitis. The udder health of nursing and non-nursing cows is reported as similar in full nursing systems [116] or similar [117,118] or better in restricted suckling or half day contact systems [68,119,120]. Passille et al., 2008 [113] suggests this is because the calf drinks residual milk soon after milking which can help prevent mastitis. The uterus of cows nursing their calf involutes faster than in non-nursing cows, potentially due to higher peripheral oxytocin in suckled cows. Since impaired fertility is one of the main reasons for culling cows prematurely at a great cost to the industry, letting cows nurse calves may improve their fertility and thus their longevity [110]. Where calves are allowed to suckle, this reduces the harvested milk yield compared to non-nursing cows and a lower milk yield can continue during the first week after weaning. However, this affect is reported to disappear in the post weaning period [113,119]. Furthermore, several studies have reported that over the whole lactation there is not a significant difference in milk yield between nursing and non-nursing cows. This has been demonstrated in a free nursing system for 1.5 week [121], a half day contact system of up to 9 weeks, and restricted suckling systems of between 3 and 26 weeks [119,122,123]. However, other studies have reported lower overall milk production so further research is required to evaluate the influence of suckling on lactation yield under commercially conditions, and the optimum age of separation for well-being and productivity.

Feasibility

Loss of saleable milk is a primary concern for farmers considering adopting management systems which allow calves to be kept with their dam beyond the first 24 hours. However, a small group of innovative farmers in the UK are trialling and marketing cow-calf dairying [124]. The daily separating and reuniting of cows and calves that is required for example in a half-day contact system is labour intensive. However, providing calves access to their dams can be controlled using automated gated systems [79,108]. Udder nets are an emerging viable intervention. A further area of consideration among some farmers is whether cow-calf rearing is detrimental to the human-animal interaction required for milking as humans no longer hand feed calves, so there is a need to

combine establishing a good human calf relationship in systems with free cow calf contact [36]. The free cow-calf contact system proves maintenance of the cow calf bond, best mimicking the natural situation, the viability of which is being put into practice here [117,121,122]. Scaling cow-calf dairying up across industry has a number of practical and production barriers for farmers, namely loss in sale of milk and not prepare the cow or calf for later separation, or a sudden change in feed or environment which can effect weight gain, or provide the opportunity for caregivers to establish a positive human-calf relationship. However, a half day contact system could provide all these benefits, as calves have the opportunity to spend time with and learn from their dam, but habituate to daily separation and management routine. A half day contact system also gives the farmers an opportunity to interact with and handle calves where learning to adapt to drink and feed from a feeder can foster nutritional independence from the dam before separation takes place [36]. A half day contact system also allows the cow to express maternal behaviours, can facilitate higher milk intake and pre-weaning growth for the calf, as well as pleasure maintenance behaviours such as play and allo grooming, and social and cognition learning from the dam [36] vital for maintaining positive affiliative relationships within the dairy herd in future.<br>Public concern about separating cow and calf immediately after birth is increasing and so is the interest and research into alternative rearing systems for dairy calves. Some of this literature was taken from a paper on the ISAE satellite workshop which concluded that 'cow calf systems can be a viable option for some producers even in our modern dairy systems' [36].
**Relevant focus group suggestions for defining "What is a good life for your cows?"**<br>No unprompted suggestions
**Summary of focus group discussion**<br>Farmers did not define positive welfare as keeping cows and calves together. This was the only opportunity unreported by the focus group in the unprompted session during the first meeting. In meeting two although farmers agreed with the value of maintaining cows and calves together from both animals' welfare, it was passionately expressed how unfeasible this practice was within the current supermarket model of maximising milk yield due to low profit margins. This opportunity was, therefore perceived to be a controversial suggestion as part of a positive welfare framework. However, it was acknowledged that the current practice of separating calves from cows soon after birth was open to criticism by animal welfare and animal rights groups, and consequently there was growing interest in this opportunity from some consumers. One member of the focus group expressed very strongly that recommending the inclusion of this opportunity in a framework on positive welfare was counterintuitive to the principle of including opportunities that all dairy farmers could provide at the Welfare + level. However, other members of the focus group felt that farmers who were already keeping calves and cows together were already being rewarded in the market place, and should be recognised by such a framework. It was also acknowledged that this was only one out of fourteen potential opportunities which dairy farmers could be recognised for. It was also articulated by the group that organic dairy farmers were already delivering this opportunity similarly to that described at the Welfare + level, apart from the practice of keeping calves in sight of the dam once separate. It was strongly expressed by the group that keeping calves in sight of the dam once separated was both hard to manage and would cause prolonged stress and frustration to both the cow and calf. It was therefore recommended that the Welfare + level be amended to synchronise Welfare + with what some organic dairy farmers were already delivering in practice which made this opportunity relevant and accessible to a wider group of dairy farmers. It was also suggested to draw a distinction between Welfare + and Welfare ++, the later should include a length of time that cows and calves are to stay together. In the absence of any research on the optimal time to separate cows and calves after allowing a bond to form but given that milk

transfer gradually declines after the first few weeks of life as calves begin to graze and ruminate, 'at least two weeks' was proposed by the facilitator. Furthermore, it was recommended that given not all dairy farmers who were delivering this opportunity were keeping calves with cows until natural weaning, an addition to Welfare +++ was made to recognise farmers who were raising calves with the dam to between 8-12 weeks of age.

Amended protocol after consideration by farmers in focus groups

Welfare + Calves are left with a lactating dam for at least two days (does not have to be the mother, can be multiple suckling on nurse cows). Calves are separated from the dam to milk feed from a teat rather than a bucket.

Welfare ++ Calves are kept with and are able to feed from a lactating dam for at least the first two weeks of life. Gradual weaning process i.e. two-step weaning used – suckling prevention then separation or a contact weaning method e.g. anti-suckling devise then fence-line contact.

Welfare +++ Calves are kept with their own dam until 8-12 weeks of age, or natural weaning. For the former, a two-step weaning process is used as described in Welfare ++.

Final data collection protocol after review from veterinary surgeons

Welfare + Are calves left with a lactating dam for at least two days? (does not have to be mother, can be multiple suckling on nurse cows)

Welfare + Are calves separated from the dam to milk feed from a teat rather than a bucket?

Welfare ++ Are calves kept with and able to feed from a lactating dam for at least the first two weeks of life?

Welfare ++ Do you use a gradual weaning process i.e. two-step weaning - suckling prevention then separation or a contact weaning method e.g. anti-suckling devise then fence-line contact?

Welfare +++ Are calves kept with their own dam until 8-12 weeks of age, or until natural weaning?

Positive Welfare Opportunity Confidence By positive experience with stock-keepers, including familiar routines and processes

Cows should be able to have positive experiences of stock-keepers and husbandry, and positive learning experiences about new people, routines, procedures and environments

Initial protocol based on scientific evidence

Law Animals shall be cared for by a sufficient number of staff who possess the appropriate ability, knowledge and professional competence.

Code It is important that grazing cattle, especially young stock come into regular contact with a stock keeper so that they will not be too frightened if they need to be gathered or treated.

Welfare +Stock keepers shall have good knowledge, attitude and practice in general dairy cow management, including flight distances and early handling [125–127]. Cows are introduced to new herd and milking parlour routines gradually and sensitively, giving opportunities to learn and adapt. Bulls receive positive attention and handling, giving opportunities to learn and adapt, for example talking, hand-feeding/ grooming (using protected contact where appropriate), training young bulls from an early age (e.g. to move around calmly when led or driven, to stand for examination).

Welfare ++: As above, **plus heifers** are introduced to new herd and milking parlour routines, stock-keepers and husbandry practices gradually and sensitively, giving opportunities to learn and adapt.

Welfare +++: As above, **plus calves** have regular positive interactions with stock-keepers from birth, over and above meeting their basic needs (feeding); for example talking to calves, brushing, gentle introduction to leg/foot and teat handling.

Validity

Particularly when kept indoors, cows rely on farmers for almost every aspect of their lives. As such, cow's daily interactions with humans have a significant impact on behaviour, well-being and productivity [128]. Where calves are separately from their dam, the human-calf relationship starts almost immediately after birth. Quick and unpredictable movements by humans evoke a fear response [129] and increase the cow's flight distance [130]. Calves who were negatively handled during the first 4 weeks of life (subject to fast movements, speaking with a harsh voice and creating noise with different tools) were less likely to approach a familiar handler compared to calves who were positively handled (i.e. moving slowly and calmly around the pen, speaking in a quiet and calm voice and encouraging positive interactions including pats and scratches) [131]. Furthermore, Lurzel et al., 2015 [132] found gentle handling within the first 14 days of life was associated with higher average daily weigh gain. Positive handling has also been associated with observer's assessment of calf behaviour. Calves handled patiently and talked to calmly were rated to be more 'friendly' and 'content,' in contrast to calves handled nervously and aggressively who were perceived to be in a more negative mood [133]. Negative interactions, such as slapping, pushing or hitting cows are negatively correlated with milk, protein and fat yields [130]. Cows will avoid handlers less in the milking parlour when they are subjected to more positive (talking quietly, stroking, and touching) and less negative interactions (using a stick or hand to move cows and shouting) [125]. Routine husbandry procedures can also be intrusive, aversive, cause pain and stress [134] and after an aversive experience, cows and calves learn to avoid that specific handler [135,136]. Cows with previous experience of feeding, stroking and calm talking (received for several minutes a day over 10 days) had a lower heart rate and kicked less when isolated for a veterinary procedure. Less restless behaviour, characterised by head shaking and tail swishing, was also associated with cows receiving positive interactions during a veterinary procedure [137]. Farmers or farm staff brushing cows manually can satisfy the want of cows to scratch and rub [138] and facilitate positive affective states, since calves were found to prefer a pen with a human that brushed them over an empty pen [139]. Furthermore, calves were observed to 'lean against the brush' and 'stretched their neck' which may indicate pleasure (although there were individual differences to calves as not all calves habituated and therefore enjoyed being brushed).

Feasibility

A means of assessing positive interactions by stock keepers has not yet been developed to measure this good life opportunity on farm. Many farmers are already achieving positive interactions with dairy calves and cows, but these aren't being formally recognised and the extent to which positive interactions are carried out may vary from staff to farm. There is likely to be a number of reasons why farmers use negative behaviour when handling and moving cows and it is important to understand the individual motives, in order to support and facilitate change. For example stock keeper fear or anxiety, frustration, negative reinforcement, temperament, attitude and the perception that negative handling is quicker or more effective are all likely to play apart. Changing any behaviour is not a quick process. It requires the individual concerned to recognise the need for and benefits of change, taking ownership over behaviour, as well as receiving a substantial amount of support and encouragement from others involved, to enable the change to be supported within the farm culture.

If farmers value and would like to focus on developing a measure to recognise positive interactions with calves and cows, the project consultation meetings should support the group to draw up interaction criteria which aim to lead to the desired outcome. To facilitate ownership over positive behaviour, the criteria and way of measuring it should be developed in collaboration with the whole farm team i.e. farm staff, advisors, veterinarian and field staff. A measuring procedure which involved each staff observing and rewarding another peer could enable positive reinforcement and peer on peer learning.

As this potential positive welfare measure requires the observation of farm staff with cows during their normal working day and over a period of time, it is not practical to be used as a means of external scrutiny, for example by a farm assurance scheme. However, if automated footage was taken and criteria were developed to grade a random sample of interactions between cows and handlers, this may become a measure of compliance in the future. But where this measure requires a grading of positive handling over time, it is more appropriate as an on farm tool conducted by and between staff (rather than a farm assurance tool). If farmers value positive social interactions between cows and caregivers, this project should support the group to develop assessment criteria with farm staff. As cows and calves interact with handlers on a day to day basis, arguably, this positive welfare opportunity has a lot of potential to enrich calves and cow's lives, and recognise and reward farmers for existing or advancing positive well-being. Furthermore, rewarding feedback from positive interactions with livestock can serve to reinforce and encourage positive behaviour and facilitate positive affective states for both the cow and caregiver.
**Relevant focus group suggestions for defining "What is a good life for your cows?"**<br>Take cows through treatment areas (procedure) without intervention or treatment (familiarity)<br>The cows own regular routine – keep disruption to a minimum<br>Provide routine complimenting their work and leisure time<br>Positive cow to stockperson interaction – quiet but interactive<br>Cows rewarded not punished for friendly rubbing<br>Expose cows to new people and experiences so they can adapt<br>Cows happy for someone to walk through them<br>Stockman personality<br>Quiet, calm atmosphere<br>Cows are happy to see people<br>Cows not scared of human interactions Easy and low stress handling<br>Early human contact
**Summary of focus group discussion**<br>In meeting one, farmers had thirteen definitions of positive welfare relating to positive experiences with stock-keepers, routines and process. The focus group expressed that stock keepers were the single most important factor in delivering positive welfare to dairy cows. In practice, farmers stated this included taking cows through a treatment area without any treatment or intervention to familiarise animals to the process, the stockpersons personality, positive cow stockperson interactions, early positive human contact, low to no flight distances and a stockperson that created a quiet, calm atmosphere while handling and managing the herd. In meeting 3 the focus group therefore agreed that providing this opportunity for positive welfare was one of the most important differences that could be made. The group made several additions and recommendations as follows. Firstly, that stock persons attitude and behaviour were emphasised at the beginning of Welfare +. There was also some discussion about the ways to positively train bulls. The farmers felt all the suggestions were of value, apart from hand-feeding which could encourage unwanted, negative behaviour towards stock-keepers.
**Amended protocol after consideration by farmers in focus groups**<br>**Welfare +** Stock keepers' behaviour towards the herd is quiet, calm, confidence, sensitive, and positive. Cows are introduced to new groups of animals, routines and the milking parlour gradually and sensitively. Positive attention, reinforcement and handling is used i.e. verbal commands, talking, hand-feeding, grooming. Cows are provided with opportunities to learn and adapt to new situations. Young bulls are trained from an early age (e.g.

to move around calmly when led or driven, to stand for examination). Bulls receive positive and gentle handling and attention (using protected contact where necessary) for training purposes i.e. talking and grooming.

Welfare ++ As above, plus heifers are introduced to new groups of animals, routines and the milking parlour with herd mates, gradually and sensitively. Positive attention, reinforcement and handling is used i.e. verbal commands, talking, hand-feeding, grooming. Heifers are provided with opportunities to learn and adapt to new situations. For example running heifers through the parlour before first lactation or integrating group of in-calf heifers with an older dry cow group.

Welfare +++ As above, plus calves have regular positive interactions with stock-keepers from birth, over and above meeting their basic needs (feeding); for example, talking to calves, brushing, gentle introduction to leg/foot and teat handling.

Final data collection protocol after review from veterinary surgeons

Welfare + Is stock keepers' behaviour towards the herd quiet, calm, confidence, sensitive, and positive?

Welfare + Are cows introduced to new groups of animals, routines and the milking parlour gradually and sensitively?

Welfare + Is positive attention, reinforcement and handling used? I.e. verbal commands, talking, hand-feeding, grooming.

Welfare + Are cows provided with opportunities to learn and adapt to new situations? Please give examples.

Welfare ++ Are heifers introduced to new groups of animals, routines and the milking parlour with herd mates, gradually and sensitively?

Welfare ++ Is positive attention, reinforcement and handling used? I.e. verbal commands, talking, hand-feeding, grooming.

Welfare ++ Are heifers provided with opportunities to learn and adapt to new situations? For example running heifers through the parlour before first lactation or integrating group of in-calf heifers with an older dry cow group?

Positive Welfare Opportunity Confidence By positive learning, resilience and social experiences within the herd

Cows confidence should be promoted by the opportunity to have positive social experiences within the herd, including establishment and maintenance of a stable social hierarchy and being able to learn about their environment from others

Initial protocols based on scientific evidence (initially 2 separate opportunities)

Good Life opportunity Confidence by positive social experiences within the herd: *Cows should be able to have positive social experiences within the herd, including establishment and maintenance of a stable social hierarchy*

Law: Feeding and watering equipment shall be designed, constructed, placed and maintained so that contamination of food and water and the harmful effects of competition between animals are minimised. Animals shall be given space appropriate to their physiological and ethological needs in accordance with established experience and scientific knowledge.

Code: The codes do not detail recommendations about establishment and maintenance of a stable social hierarchy. The accommodation should provide enough space for a subordinate animal to move away from a dominant one. You should carefully introduce dairy heifers to the adult herd at least four weeks before calving.

Welfare + Adult dairy cows are housed and kept in such a way that they can establish and maintain a stable social hierarchy within the herd. Farm conditions enable and maintain improved group cohesion, building or strengthening of bonds between group mates and reduced aggression [18,60,140,141].

Welfare ++: As above, plus heifers are batch-reared in such a way that they can establish and maintain a stable social hierarchy and this is maintained into the adult milking herd.

Welfare +++: As above, plus calves are batch-reared in such a way that they can establish and maintain a stable social hierarchy and this is maintained throughout their lives [140]. A bull runs with the herd (may be vasectomised).

AND

Good Life opportunity Confidence by learning and resilience gained from dam and mixed age group: *Cows should be able to learn about their social and physical environment from other cows and become resilient to changes in husbandry, management and social structure*

Law: The law does not detail requirements about learning and resilience gained from dam and mixed age group.

Code: You should carefully introduce dairy heifers to the adult herd at least four weeks before calving.

Welfare + Calves/ young stock are kept and reared together in pairs or groups from the time they are separated from the dam [75,142–146].

Welfare ++ As above, plus calves/ young stock have opportunities to learn appropriate herd social behaviour within familiar mixed age groups [80,147,148].

Welfare +++ As above, plus calves/ young stock/ heifers have opportunities to learn milking and stock-keeping routines and processes gradually alongside familiar, experienced cows/ cohorts [65,79,81,147]. For example, running younger animals through the parlour with the milking herd before their first lactation.

Validity

Dairy cows are a social herd species. Positive social experiences within the herd improves biological functioning, helps animals cope with stress (social buffering) [142,149], reduces frustration and aggression [141], improves social and cognitive development [95] and increases behavioural opportunities which lead to positive affective states [18]. When given the opportunity, calves are motivated to get full bodily access to another calf, compared to head contact through metal bars [150]. Calves raised in full contact of other calves form strong bonds which affect their social preferences throughout adulthood [143,144]. Individual housing of calves' stunts development and feeding behaviour. In contrast, calves housed in pairs visit the feeder quicker and spend more time at the feeder [75]. Individual rearing also reduces the calf's ability to cope with environmental stressors, reduces social skills [142,151] and increases heart rate during confrontation [152]. Furthermore, the stress of weaning can be reduced by pair or group housing of calves. Pair or group housed calves were found to vocalise less when weaning [75]. The stress of isolation can also be reduced by a companion, and in particular a familiar companion [145]. Individually reared calves spend more time alone and have a lower social rank when introduced to a new group compared with group housed calves [153]. More recently, it was found that calves that have had the opportunity to interact with other cows are more resilient and flexible to changes in management and housing [95]. In intensive dairying, milking cows are grouped and regrouped according to their physiological and milk production status which interferes with the social dynamic of the group and inhibits the maintenance of affiliative relationships. Regrouping leads to increased physical competition in the hours and days afterward it takes place, as well as reducing milk production [141]. Introducing a cow to a new group also reduces their feeding and lying time, and occurrence of allogrooming. Keeping cows in stable social groups would provide heifers with the opportunity to maintain affiliative bonds. These benefits include social companionship, social cohesion and social buffering [154]. Social buffering, which serves to help relieve stress is expressed through grooming behaviour. Allogrooming is an indicator of friendship [18], serves to reduce conflict [65] and promotes a calm affective state for the receiver [64]. Where maintaining a continuous stable social hierarchy throughout a cow's life is not feasible, regrouping with a friend they have been reared with is currently seen as the next best

possible means of promoting positive social experiences and resilience within the herd. Fewer antagonistic interactions and greater synergy between new heifers and the new groups lying and feeding behaviour is seen when heifers are introduced with a friend [155,156].

Feasibility
A means of assessing the good life opportunity of confidence through positive social experiences, learning and resilience from the dam and within a mixed age group has not yet been developed for on farm use. The feasibility and palatability of considering these positive welfare opportunities are likely to vary depending on the ethos and size of the farm. However, many farmers are already group rearing dairy calves and may see the value of extending the opportunity for keeping cows that are friends together or better still more of a stable social hierarchy throughout the cow's life. It would be useful to develop a measure to assess the impact of management changes which promote positive social experiences, learning and resilience within the herd, such as monitoring the behaviour of group or pair reared calves and heifers. If farmers who are part of the consultation meetings see the value in recognising stable social hierarchy for their dairy herds, this project should support the group to develop a measure to recognise the added positive welfare.

Pair or small group housing of calves has economic benefits for the farmer since it requires less space than individual housing, allowing more space per group to a) facilitate play behaviour and b) provide more space between groups to reduce disease transmission [138]. Practical considerations of feeding in group housed calves to reduce competition and cross-suckling behaviour can be addressed by providing a nipple to drink milk through and a barrier between feeding stations [138]. However, maintaining a stable social hierarchy for cows throughout life is less practical as herd size increases and intensifies. However, rearing calves in pairs (if not groups) and always providing heifers and cows with a friend when being introduced to a new group is the next best practical option. As keeping calves in such a way to maintain stable social hierarchies is a management style and resource which can be visually assessed on farm, for this age group, this maybe a measure for consideration as part of farm assurance. And where criteria are established and can be discussed and verified by the farmer, this may also be applicable for the cow groups.

Relevant focus group suggestions for defining "What is a good life for your cows?"
Social experiences :
Positive cow to cow interaction
Timid heifers not allowed to be bullied
Cows not eating on their own which would indicate they are being bullied
Routes around the shed, especially for quiet cows and heifers
Measure confidence within the herd
Lots of space for positive social interactions
Avoid bullying by dominant cows
Avoid mixing groups so each cow has a place in the hierarchy
Respect social hierarchy
Learning experiences :
Not changing social groups
Respect social hierarchy

Rearing calves together so they have a social group<br>Calving into the herd at the same time so can stay in herd together<br>Social grouping – minimise group changes or move several 'friend' cows together<br>Having a group of cows that can get to know each other
**Summary of focus group discussion**<br>In meeting one, farmers reported nine definitions of positive welfare relating to confidence by positive social experiences within the herd (see above). These consisted of maintaining a stable herd, avoiding mixing groups, providing lots of space to give all cows choice and to facilitate positive social interactions. The focus group also articulated six definitions relating to confidence by learning and resilience gained from dam and mixed age group. In meeting 3 the farmers discussed the similarities between these two opportunities and decided there was not enough distinction to warrant separating them. Therefore, a new opportunity was created 'confidence by positive learning, resilience and social experiences within the herd which was put forward for recommendation.
**Amended protocol after consideration by farmers in focus groups**<br>Two opportunities combined into one<br>**Welfare +** Cows are kept in such a way that they can establish and maintain a stable social hierarchy within the herd (Maintaining group cohesion, bonds and reducing aggression).<br>**Welfare ++** As above, plus young stock (6 months+) are group-reared (from weaning) in such a way that they can establish and maintain a stable social hierarchy within the group which is maintained into the adult milking herd.<br>**Welfare +++** As above, plus calves (up to 6 months) are kept and reared together in groups from the time they are separated from the dam, in such a way that they can establish and maintain a stable social hierarchy which is maintained throughout their lives. A bull runs with the herd (may be vasectomised) allowing natural behaviour to be expressed and natural social hierarchy and herd stability to be maintained.
**Final data collection protocol after review from veterinary surgeons**<br>**Welfare +** Are cows kept in such a way that they can establish and maintain a stable social hierarchy within the herd? (Maintaining group cohesion, bonds and reducing aggression).<br>**Welfare ++** Are young stock (6 months+) group-reared (from weaning) in such a way that they can establish and maintain a stable social hierarchy within the group?<br>**Welfare ++** Is this maintained into the adult milking herd?<br>**Welfare +++** Does a bull run with the herd (may be vasectomised) allowing natural behaviour to be expressed and natural social hierarchy and herd stability to be maintained?
**Positive Welfare Opportunity Interest** By a positively enriched environment<br>*Cows should be able to experience positive emotional states through cognitive enrichment including novelty and exploration*

Initial protocol based on scientific evidence

Law The law does not detail requirements about positive enrichment including novelty and exploration. Animals shall be given space appropriate to their physiological and ethological needs in accordance with established experience and scientific knowledge.

Code The codes do not detail recommendations about positive enrichment, including novelty and exploration.

Welfare +: Opportunities for positive contrast situations (such as choice between two or more positive activities) and for exploration (such as toys, puzzles and tasks) [18,157–159] are provided occasionally throughout life (for example hay balls, cardboard boxes, brushes, padded posts, sand pit).

Welfare ++: As above, but provided continuously throughout life.

Welfare +++: As above, but provided continuously and changed frequently throughout life.

Validity

Cognitive enrichment has played an important role in advancing the well-being of pigs, but these opportunities have not been realised to the same extent for dairy cows [138]. Housing offers less opportunity for cows to use their cognitive abilities and explore their environment for food as they would in the wild [160]. Providing cow's with choices of interest, such as resources to explore tactilely, food to find, brushes for self-grooming or problem solving leading to a reward particularly while housed, can improved biological functioning, help prevent frustration, act as a buffer to reduce stress and increase the repertoire and expression of behaviour which lead to positive affective states [138]. Cognitive enrichment can give cows control over their environment and lead to positive affective states. Housed cows spend far less time feeding compared to cows at pasture (4 hours v. between 6 and 12 hours) due to the fact there is no need to search for their food and its easy consumed form. Appetitive or seeking behaviours expressed while exploring a toy, puzzle or task are associated with dopamine release, and consummatory behaviours of receiving a food reward are associated with opioids [161]. Manteuffel et al., 2009 [162] suggested that cognitive enrichment can be provided using self-controlled operant learning tasks. Hagan and Broom 2004 [163] found heifers that learnt to open a gate to gain a food reward had higher heart rates and more vigorous movement due to the excitement of understanding and gaining control over the task. Providing part of the daily food ration in a puzzle or hay net can also provide cows with the opportunity to work or explore for their food. Dairy calf and cow housing can be enriched by changing the size or complexity of the indoor housing area with new objects, substrates or structures. Breaking up the indoor housing for different functions encourages exploration and patrolling by calves, as well as camouflage and hiding [164]. Furthermore, enriching the environmental with brushes and log piles and breaking up the space with a wooden wall had the benefit of reducing antagonistic interactions between calves [165]. Provision of substrates such as straw may also be associated with play behaviour in calves [152]. Similarly, brushes and balls have been found to encourage calf play [166]. In the wild and at pasture, cows use trees and other objects to self-groom. In the absence of outdoor access, cows are seen rubbing their head, neck and body on available housing objects, such as water troughs, metal fences and gates [167]. Cows show a clear preference to use brushes. Brushes installed in housing are found to be used daily by calves, cows and breeding bulls [168] and are associated with better body cleanliness and improved milk yield in second lactation [169]. Furthermore, brush use may be seen as an indicator of a positive welfare state since cows use brushes less when they are ill or stressed [170].

Feasibility

While cows are housed and certainly in the case of cows who are continually housed, providing cognitive, physical and sensory enrichment can provides cows with the opportunity to express more of their natural

behavioural repertoire in an otherwise confined and relatively barren environment. Farmers are likely to be quite motivated to adopt some of these interest opportunities, as, in comparison to other good opportunities, enriching the environment is fairly inexpensive to implement and does not require major cultural management changes. These opportunities also have the benefit of being easily assessed by farmers and farm assurance bodies and the use of enrichment within the cow's environment can be manually or automatically monitored. With an increase in intensification and permanently housed cattle, enriching the indoor environment is going to become more important to allow cows to express a wider range of behavioural outcomes which can lead to positive affective states.
**Relevant focus group suggestions for defining "What is a good life for your cows?"**<br>Playing with or exploring a piles of dirt/substrate<br>Brushes – our cows queue for them so we know they like them<br>Having a rub on a hedge or a tree
**Summary of focus group discussion**<br>Farmers described three definitions of positive welfare relating to positively enriching the environment in meeting 1 (see above). In focus group meeting 3, farmers agreed with the principle of interest by a positively enriched environment and added a number of existing ways in which interest is created by enriching the indoor and outdoor environment (Welfare +).
**Amended protocol after consideration by farmers in focus groups**<br>**Welfare +** Cows are provided with opportunities for choice between two or more positively stimulating activities, provided **sometimes** throughout life (e.g. hay balls, cardboard boxes, brushes, padded posts, sand pit, hay bales, tree stumps, hedge rows, tractor tyres, music).<br>**Welfare ++** Cows are provided with opportunities for choice between two or more positively stimulating activities are provided continuously.<br>**Welfare +++** Cows are provided with opportunities for choice between two or more positively stimulating activities are provided continuously and changed or replenished frequently (i.e. between 2 weeks and 3 months).
**Final data collection protocol after review from veterinary surgeons**<br>**Welfare +** Are there opportunities for choice between two (or more) positively stimulating activities, provided sometimes throughout life? (E.g. hay balls, cardboard boxes, brushes, padded posts, sand pit, hay bales, tree stumps, hedge rows, tractor tyres, music).<br>**Welfare ++** Are there opportunities for choice between two (or more) positively stimulating activities, provided continuously throughout life?<br>**Welfare +++** Are there opportunities for choice between two (or more) positively stimulating activities, provided continuously and changed or replenished frequently throughout life? (I.e. between 2 weeks and 3 months).
**Positive Welfare Opportunity Interest** By pasture choices<br>*Cows should be able to exercise individual preferences about their access to and use of pasture at all times*
**Initial protocol based on scientific evidence**<br>**Law** The law does not detail requirements about access to pasture. The freedom of movement of animals, having regard to their species and in accordance with established experience and scientific knowledge, shall not be restricted in such a way as to cause them unnecessary suffering or injury.

Code The codes do not detail requirements about access to pasture.
Welfare + Choice to have access to pasture during parts of the day/night during summer season [171–178].
Welfare ++ Choice to have access to pasture at all times of day/night during the summer season [171,172,175,178–182].
Welfare +++ Choice to have easy access to sheltered pasture at all times of day/night in all seasons (sufficient ease of access and shelter to enable safe use of pasture in winter).

Validity
Cows on pasture-based systems generally have lower levels of lameness, hoof pathologies, hock lesions, mastitis, uterine disease and mortality compared with cows on continuously housed systems. Pasture access also has benefits and opportunities for dairy cow behaviour, such as roaming, grazing, exercise, improved lying/resting times and lower levels of aggression. Moreover, when given the choice between pasture and indoor housing, cows show an overall preference for pasture, particularly at night. Lameness is a major health and welfare problem. The cost to the industry is second only to mastitis, reducing feeding time, is associated with lower body condition scores, reduces milk yield and has a substantial negative effect on fertility and can lead to premature culling. Lameness is multifactorial but access to pasture certainly has a protective effect [173,183]. Grazing cows were far less likely to be clinically lame (17% v. 61% prevalence) from day 180 post calving onwards [173]. Furthermore, a significant reduction in clinical lameness in cows at pasture was found within a 4 week period [183]. Therefore, protective effect of grazing can be seen quickly but the benefit is also long term. Haskell et al., 2006 [175] found a significantly lower prevalence of lameness during the winter when all cows were housed on farms that allowed grazing during the summer compared to continuously housed cows (15% v. 39% prevalence). Housing is also associated with an increase in all infectious or traumatic hoof diseases and this affect increases significantly with time, and carries over into the pasture period [173,184]. The positive benefits of access to pasture are thought to be a natural, comfortable, soft and hygienic standing and walking surface [174,185], as well as promoting exercise [76,185], reducing restlessness and increasing lying times [173]. Pasture access also reduces hock lesions [186–188].

Pasture is also seen to have a protective affect against mastitis, one of the largest cost to the dairy industry. Pasture based cows have a reduced prevalence of mastitis (31% v. 51%), number of cases per cow (0.6 v. 1.1), and less are culled (1.6% v.9.7%) [176,177]. This effect is so far thought to be due to less exposure of environmental pathogens and increased cow cleanliness (although summer mastitis is likely to be more of a risk in pasture based systems). Less dystocia, metritis and endometritis has also been found in pasture based systems [174]. Unrestricted grazing during summer had a protective effect against salmonellosis [189]. Furthermore, herds were more likely to be in a high mortality group if cows were not on pasture during the summer season [190].
Kilgour [191] reviewed 22 studies of feral cattle (free living cows at pasture with little human contract) and identified grazing as the most common behaviour, followed by ruminating and resting, accounting for 90% to 95% of the cows day. Most grazing is performed during daylight and cattle spend more time resting and ruminating at night. There is also diurnal rhythm of behaviour, characterised by peaks of grazing activity associated with sunrise and sunset. Cows on pasture spend more time feeding (68% v. 22%), have more synchronised feeding behaviour and similar time budgets to feral cows [191,192]. Cows have greater mean total lying times per 48 h period (42.7% v. 37.7% of time spent lying) and longer lying bouts (50.3 v. 39.3 min) [173] and more synchronised lying on pasture [193]. Lying deprivation is physiologically stressful and can be associated with elevated cortisol levels, reduced adrenocorticotrophic and cortisol responses following

corticotrophin releasing hormone challenge [194]. It has also been observed that cows on pasture have reduced aggression as there is no competition over food and far more space. O'Connell et al. [193] reported that agonistic interactions occurred at low levels at pasture and were significantly greater in housing, peaking around the delivery of food.

The best way to know what animals want is to give them the choice and see what they prefer. In Canada, Falk et al., [195] found cows showed a partial preference for pasture (57% of their time) with more time spent outside at night (78.5%) than during the day (41.5%). In the UK, Charlton [179] gave cows the choice between indoor cubicle housing (with access to TMR) and pasture (with half of the trials also offering TMR on pasture to see how this influenced the choice) and found cows spent more time on pasture than indoors (71% v. 29%), with more time spent on pasture at night than during the day (84% v. 51%). Furthermore, TMR offered on pasture did not increase pasture use. In addition, the motivation to use pasture appears to differ depending on time of day. Motupalli et al., [196] found cows spent more time at pasture in the near (38 m) compared with far distance (254 m), but this had no effect on pasture use at night. Overall, studies so far suggest that during the day, cows have a partial preference for indoor housing [171,180,195] or spend or spent similar time periods indoors and on pasture [179,196]. However, at night cows prefer to be at pasture [179,180,195] and are particularly motivated to be so since they will walk further for this opportunity [171,196].

The behavioural benefits of access to pasture must be balanced by the physiological hazards. Since pasture preference reduces with increased rainfall [179,180,195] humidity [179,180,195] and season [179], it is important to provide cows with adequate shelter, ideally trees, which offer the addition benefit of grooming objects. Wet and windy conditions reduces lying time significantly (4 hours compared to 12 hours) [52,56], and can increase cortisol levels and immunosuppression where thinner cows are particularly susceptible [56,197]. Cows will use microclimates that provide protection [198], including sheltering along hedge rows and under trees to mitigate physiological stress. These resources are imperative to maximise the benefits of access to pasture. In addition, trees can act as a biosecurity buffer between herds and farms.

Cows have access to sunlight at pasture [199]. Sunshine is an important source of Vitamin D and Vitamin D status is associated with time spent on pasture during the summer [200]. Biomedical research has found that sun exposure has positive effects on cardiovascular health, lowering blood pressure [201] and on immune function [202]. Furthermore sunlight exposure is rewarding, with hedonic and addictive properties [203]. How sunlight may induce positive emotional states in dairy cows with outdoor access should be subject of further investigation.

A recent UK study suggests that only 31% of dairy farms now maintain a traditional grazing system (with no summer forage fed indoors), and 8% of farms housed milking cows all year, and high yielding or early lactation cows are continually housed on a further 8% of farms [204]. This trend towards further intensification is in conflict with the good life opportunity of interest for cows to have the choice of access to pasture. The research literature summarised here demonstrates a host of not only behavioural but also a plethora of health benefits of access to pasture. Furthermore, a survey in Britain found that 95% of consumers that were questioned did not think it was acceptable to keep dairy cows permanently indoors [205]. And more recently, pasture access was viewed as important for welfare amongst those who were both affiliated and unaffiliated with the dairy industry in North America [206]. The European Food Safety Authority (EFSA) [207] states that 'when possible, dairy cows and heifers should be given access to well managed pasture or other suitable outdoor conditions, at least during summer or dry weather.'

Relevant focus group suggestions for defining "What is a good life for your cows?"

Loads of loafing area/paddock to express behaviour

The opportunity to go outside and graze

Summary of focus group discussion

At the first focus group, farmers defined positive welfare as loads of loafing area or paddock for cows to express natural behaviour and the opportunity to go outside and graze. During meeting 3, farmers discussed the draft proposal and feasibility of providing cows with a choice of access to pasture and concluded this was impractical for the vast majority of farmers. Therefore this opportunity was recommended to reflect the best possible existing management strategies with regards to an increase in the time cows spent at pasture, including an aspirational addition to Welfare +++ of providing access to shade and shelter for all animals at all times when under an extended grazing system.

Amended protocol after consideration by farmers in focus groups

Welfare + Cows have access to pasture during parts of the day/night during the summer grazing season.

Welfare ++ Cows have **continuous access** to pasture during the summer grazing season with access to shade and shelter.

Welfare +++ Cows have continuous access to sheltered pasture for an extended grazing seasons i.e. at least 200 days a year. There are sufficient trees, hedgerows, or artificial shelter/shade for all cows to shelter from undesirable weather at the same time.

Final data collection protocol after review from veterinary surgeons

Welfare + Do cows have access to pasture during parts of the day/night during the summer grazing season?

Welfare ++ Do cows have continuous access to pasture during the summer grazing season?

Welfare +++ Do cows have continuous access to sheltered pasture for an extended grazing season i.e. at least 180-200 days a year? +++ Are there sufficient trees, hedgerows, or artificial shelter/shade for all cows to shelter from undesirable weather at the same time?

Positive Welfare Opportunity Interest By food choices

Cows should be able to exercise individual preferences for type of food and how it is obtained

Initial protocol based on scientific evidence

Law Animals shall be fed a wholesome diet which is appropriate to their age and species and which is fed to them in sufficient quantity to maintain them in good health and to satisfy their nutritional needs and to promote a positive state of well-being.

Code All cattle need a balanced daily diet to maintain full health and energy. You should monitor how much forage is available and when there is no longer enough for the animals' needs, you should supplement it with other suitable feeds. Sufficient roughage must be available in all diets to reduce the risk of inducing bloat or laminitis.

Welfare + From weaning, animals are offered a varied diet both indoors and at pasture. For example, at least two types of forage (silage, hay, straw); turnips or fodder beet; poorly mixed TMR which enables some selection of preferred components.

Welfare ++ As above, plus animals are offered a choice of pasture herbage (such as clover plus mixed sward) (Rutter 2010) and, if provided, a choice of forage/ concentrate rations.

Welfare +++ As above plus the choice is varied at intervals over time

**Relevant focus group suggestions for defining "What is a good life for your cows?"**<br>Allowing cows to eat when they want<br>Good access to what they desire to eat for all<br>Never waiting for food or water<br>Satiety - food satisfaction
**Summary of focus group discussion**<br>Farmers articulated four ways positive welfare can be delivered with food provision. The focus group agreed with the principle of interest by food choices but were concerned that the wording of Welfare + was not clear enough in explaining that opportunities for choice should come in additional to making sure cows receive a sufficient amount of nutritionally balanced diet. The farmers stated that the aims of this opportunity were not clear enough, and that it was more practical to separate food choices in the indoor environment (Welfare +) with food choices in the outdoor environment (Welfare ++ and +++) to reflect how cows were managed during the course of the year . Finally, Welfare +++ was neither deemed valuable or feasible enough to warrant a point of distinction.
**Amended protocol after consideration by farmers in focus groups**<br>**Welfare +** Cows are provided with some variety in their diet indoors above and beyond a sufficient amount of nutritionally balanced diet i.e. at least two types of forage (silage, hay, straw, barley); turnips or fodder beet, coarsely mixed TMR and/or food presented in different/novel places/ways to facilitate exploration and seeking behaviour.<br>**Welfare ++** Cows offered a choice of pasture herbage outdoors (such as clover plus mixed sward). Cows are provided with a choice of forage/concentrate rations in the field, or are presented in different/novel places/ways.
**Final data collection protocol after review from veterinary surgeons**<br>**Welfare +** Are cows provided with some variety in their diet indoors above and beyond a sufficient amount of nutritionally balanced diet? I.e. at least two types of forage (silage, hay, straw, barley); turnips or fodder beet, coarsely mixed TMR and/or food presented in different/novel places/ways to facilitate exploration and seeking behaviour.<br>**Welfare ++** Are cows offered a choice of pasture herbage outdoors (such as clover plus mixed sward)?<br>**Welfare ++** Are cows provided with a choice of forage/ concentrate rations in the field, or are these presented in different/novel places/ways?
**Positive Welfare Opportunity Healthy Life** By the stockpersons knowledge of individual cows' habits and references<br>*Cows should be able to have their individual needs and choices met through good stock-keeping*
**Initial protocol based on scientific evidence**<br>**Law** All animals kept in husbandry systems in which their welfare depends on frequent human attention shall be thoroughly inspected at least once a day to check that they are in a state of well-being. Animals kept in systems other than husbandry systems in which their welfare depends on frequent human attention shall be inspected at intervals sufficient to avoid any suffering.<br>**Code** All stock-keepers should be familiar with the normal behaviour of cattle and should watch for any signs of distress or disease.<br>**Welfare +** Stock-keepers recognise and investigate changes in behaviour within 24 hours. Stock-keepers know the individual habits and preferences of **some** cows in the herd [60,208].

**Welfare ++** As above but stock-keepers know the individual habits and preferences of **most** cows in the herd [60,126,140].<br>**Welfare +++** As above but stock-keepers recognise, investigate **and act on** changes in behaviour within 24 hours. Stock-keepers know the individual habits and preferences of **every** cow in the herd [60,125,126].
**Relevant focus group suggestions for defining "What is a good life for your cows?"**<br>Giving cows space to express their individual personality<br>Knowing all your cows – for example boss cow not in her usual place tells you something is up<br>Scope for cows to be themselves<br>Knowing your animals individual habits
**Summary of focus group discussion**<br>Four definitions of positive welfare in relation to stockpersons knowledge of cow habits, preference and personalities were articulated at the first focus group meeting (see appendix 4). The focus group expressed that contrary to Welfare + this opportunity was only adding value and delivering positive welfare opportunity when the stock people knew the individual habits, preferences and personalities of most (Welfare ++) or all (Welfare +++) of the herd. Therefore the group proposed to change the wording to recommend two levels.
**Amended protocol after consideration by farmers in focus groups**<br>**Welfare +** Stockpersons know the individual habits and preferences of **most** cows in the herd. Stockperson recognise and investigate changes in cow behaviour for health and welfare reasons within 24 hours. Stockperson recognises and investigates changes in behaviours, such as a cow in a different position in the milking parlour, body language/demeanour, activity/energy level, welfare outcome i.e. lameness, or other.<br>**Welfare ++** Stockpersons know the individual habits and preferences of **every** cow in the herd
**Final data collection protocol after review from veterinary surgeons**<br>**Welfare +** Do stock-keepers know the individual habits and preferences of some cows in the herd? + Do stock keepers recognise and investigate changes in cow behaviour for health and welfare reasons within 24 hours? + What changes of behaviour do you recognise and investigate? Such as cow in different position in milking parlour, body language/demeanour, activity/energy level and welfare outcome i.e. lameness<br>**Welfare ++** Do stock-keepers know the individual habits and preferences of every cow in the herd?
**Positive Welfare opportunity Healthy life** By effective management of day to day health and welfare<br>*Stockpersons manage day-to-day dairy cow health and welfare*
**Initial protocol based on scientific evidence**<br>**Law** Animals which appear to be ill or injured shall be cared for appropriately without delay. Where they do not respond to such care, veterinary advice shall be obtained as soon as possible.<br>**Code** The stock-keeper should draw up a written health and welfare plan with the herd's veterinary surgeon and, where necessary, other technical advisors, which should be reviewed and updated each year. This plan should set out health and husbandry activities that cover the whole year's cycle of production, and include strategies to prevent, treat or limit existing disease problems. The plan should include records to enable you to monitor and assess the health and welfare of the herd.<br>**Welfare +** The health and welfare programme should be implemented and reviewed frequently plus action taken to reduce or alleviate the cause of any health and welfare problems. Routine use of medicines and mutilations should not be substitutes for good management. Stock-keepers manage vulnerable groups (e.g. freshly-calved heifers, high-yielding cows) with attention and positive management strategies for their group needs.

**Welfare ++** As above, plus regular dialogue with veterinarian and scheme welfare advisor. Stock-keepers manage vulnerable individual animals (e.g. freshly-calved heifers, high-yielding cows) with attention and positive management strategies for their individual needs.<br>**Welfare +++** As above, plus stock-keeper takes active part in welfare activities with wider benefits (e.g. member of scheme policy/ management group, peer advisor, on-farm welfare research).
**Relevant focus group suggestions for defining "What is a good life for your cows?"**<br>Outward stockperson who regularly observes the cows and herd overall appearance – i.e. nutritional fullness, cleanliness/shininess of coat, no coat or body damage/injury free<br>Proactive health management to improve herd health i.e. biosecurity, vaccinating for diseases, removing persistently diseased animals<br>Good welfare outcomes – i.e. clean cows
**Summary of focus group discussion**<br>During meeting 1, the focus group reported in their own words three ways in which cow's healthy life can be delivered by effective management of day to day health and welfare. On discussing the draft for this opportunity at meeting 3, the farmers expressed that in order to warrant a clear distinction between Welfare + and Welfare ++, a requirement for proactive ongoing welfare training should be included, along with the recommendation (previously highlighted) that stock people at this level should be regularly using manual or automatic systems to monitor the impact of resources (see Welfare ++). Finally, farmers suggested that a demonstration of active participation in welfare improvement activities in Welfare +++ could also be achieved by taking part in welfare discussion groups, or is a welfare showcase/host farm.
**Amended protocol after consideration by farmers in focus groups**<br>**Welfare +** Stockpersons implement and review a health and welfare action plan frequently. Action is taken immediately to reduce or alleviate the cause of any health and welfare problems. The routine use of antibiotics/medicines is being actively reduced. Vulnerable groups are managed differently with strategies for their needs<br>**Welfare ++** Stock people work proactively with their vet (and other support actors i.e. consultant or scheme advisor) to improve welfare. Stock people take part in proactive ongoing welfare training. The farm actively monitors welfare using cameras, activity meter data, behavioural observations, qualitative behavioural assessment, cow signals and/or welfare outcome assessment. The farm uses this data to monitor and adjust resources and/or management in improve positive welfare experiences accordingly.<br>**Welfare +++** Farm takes part in welfare improvement activities with wider benefits i.e. member of welfare policy/advisory group, peer advisor, welfare discussion groups, participates in on-farm welfare research or innovation, or is a welfare showcase/host farm.
**Final data collection protocol after review from veterinary surgeons**<br>**Welfare +** Is a health and welfare plan implemented and reviewed frequently?<br>**Welfare +** Is action taken immediately to reduce or alleviate the cause of any health and welfare problems?<br>**Welfare +** Is the routine use of antibiotics/medicines being actively reduced?<br>**Welfare +** Are vulnerable groups managed differently with strategies for their needs?<br>**Welfare ++** Does the farm work proactively with their vet (or other support actors i.e. scheme advisor)?<br>**Welfare ++** Do stock-keepers take part in proactive ongoing welfare training?<br>**Welfare ++** Does the farm monitor welfare using cameras, activity meter data, behavioural observations, cow signals and/or welfare outcome assessment?

**Welfare ++** Does the farm use this data to monitor and adjust resources to improve welfare accordingly?<br>**Welfare +++** Do farm staff take part in welfare improvement activities with wider benefits? I.e. member of welfare policy/advisory group, peer advisor, welfare discussion groups, participates in on-farm welfare research/innovation.
**Positive welfare Opportunity Healthy Life** By positive genetic selection for long-term health and welfare<br>*Stock keepers should influence long term health and welfare of the dairy herd*
**Initial protocol based on scientific evidence**<br>**Law** No animals shall be kept for farming purposes unless it can reasonably be expected, on the basis of their genotype or phenotype that they can be kept without detrimental effect on their health or welfare.<br>**Code** A high priority in the breeding selection policy should be to include qualities that will improve the welfare of the animals. You should not breed from any animals that have deformities or other weaknesses, where these could affect the general welfare of the stock.<br>**Welfare +** Farm manager recognises undesirable side effects of genetic selection for production efficiency [209–211] and selects sires and replacement animals to reduce/ mitigate for current health issues within the herd e.g. lameness, mastitis, metabolic disease, anxiety/ reactivity [212].<br>**Welfare ++** As above, plus farm manager makes choices for potential future health and welfare issues within the herd, valuing these equally to milk yield and other production factors.<br>**Welfare +++** As above, plus farm manager chooses replacement animals for long-term improvement of herd health, welfare, resilience and metabolic normality, valuing these over milk yield and other production factors.
**Relevant focus group suggestions for defining "What is a good life for your cows?"**<br>Use genetic indices and genomics, available for bulls and for genomic testing of replacement heifers before breeding and rearing for traits such as lifespan, fertility, feet, legs, somatic cell counts<br>Use of polled bulls to avoid need to disbudding. If 2 bulls are very similar for all other traits, opt for polled one good positive welfare choice
**Summary of focus group discussion**<br>At meeting group 1, one farmer provided two practical examples of how to deliver healthy life by positive genetic selection for long term health and welfare in the free choice exercise on positive welfare definitions. At the third meeting, this opportunity was discussed in detail where the focus group proposed several additions to the Welfare + level, namely using polled bulls to avoid disbudding (where all other traits are the same) and using genomic testing of replacement heifers for additional traits of lifespan and resilience.
**Amended protocol after consideration by farmers in focus groups**<br>**Welfare +** Sires/replacement animals are selected for breeding with the aim to reduce current health/welfare issues within the herd e.g. lameness, mastitis, metabolic disease (for example Immunity +), lifespan, resilience/robustness, temperament, reduced anxiety/reactivity, reduced need to mutilate i.e. using polled bulls to avoid disbudding. Bulls are selected on health and welfare traits.<br>**Welfare ++** Breeding choices for future herd health and welfare valued equally to breeding choices made for milk yield and other production factors.<br>**Welfare +++** Breeding choices for future herd health and welfare valued more than breeding choices made for milk yield and other production factors.
**Final data collection protocol after review from veterinary surgeons**<br>**Welfare +** Are sires/replacement animals selected for breeding with the aim to reduce current health/welfare issues within herd e.g. lameness, mastitis, metabolic disease (for example Immunity +), lifespan,

resilience/robustness, temperament, reduced anxiety/reactivity, reduced need to mutilate i.e. using polled bulls to avoid disbudding? + Are bulls selected on health and welfare traits?

Welfare ++ Are breeding choices for future herd health and welfare valued equally to breeding choices made for milk yield and other production factors?

Welfare +++ Are breeding choices for future herd health and welfare valued more than breeding choices made for milk yield and other production factors?

Table S2 Policy development process (Adapted from [29])

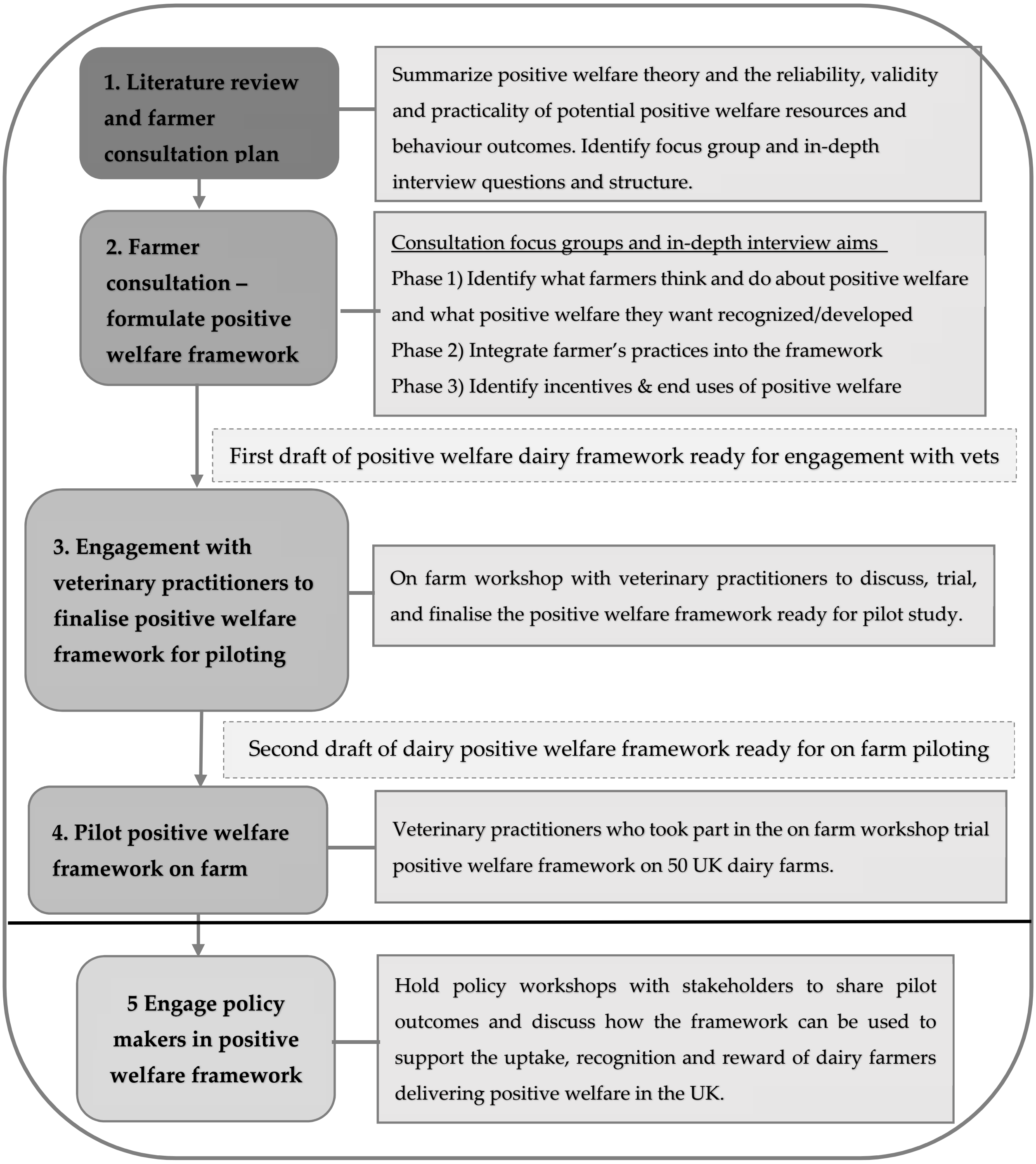

Supplement: Supplementary file 1 [file animals-12-02540-s001.zip › animals-1858426-supplementary (5).pdf]
